# Comparing optimal transport and machine learning approaches for databases merging in scenarios involving missing data in covariates. Application to medical research

**DOI:** 10.1093/bioinformatics/btag594

**Published:** 2026-08-08

**Authors:** Flore N’kam Suguem, Sébastien Déjean, Philippe Saint Pierre, Nicolas Savy

**Affiliations:** Institut de Mathématiques de Toulouse, UMR521, Université de Toulouse, CNRS, Toulouse, France; Université de Toulouse UT, Toulouse Cedex 9 F-31062, France; Department of Physics and Astronomy, University of Bologna, Bologna 40127, Italy; Institut de Mathématiques de Toulouse, UMR521, Université de Toulouse, CNRS, Toulouse, France; Université de Toulouse UT, Toulouse Cedex 9 F-31062, France; Institut de Mathématiques de Toulouse, UMR521, Université de Toulouse, CNRS, Toulouse, France; Université de Toulouse UT, Toulouse Cedex 9 F-31062, France; Institut de Mathématiques de Toulouse, UMR521, Université de Toulouse, CNRS, Toulouse, France; Université Toulouse Jean-Jaurès UT2J, Toulouse F-31058, France

## Abstract

**Motivation:**

One of the challenges encountered when merging heterogeneous observational medical datasets is the recoding of categorical target variables that may have been measured differently across data sources. This study compares standard machine learning-based approaches, specifically Multiple Imputation by Chained Equations (MICE), *k*-Nearest Neighbours (kNN), missForest, and Factor Analysis of Mixed Data (missMDA), with an Optimal Transport-based algorithm (OTrecod). Their empirical performance is evaluated in realistic data integration settings that incorporate missing covariate values, non-linear relationships, and imbalanced groups, all of which remain underexplored.

**Results:**

A comprehensive simulation study was conducted, varying sample size, group imbalance, signal-to-noise ratio, non-linear constraints, and missing data mechanisms and proportions. The results reveal two distinct performance tiers, with missForest, missMDA, and OTrecod often outperforming MICE and kNN. While missForest achieves the highest recoding accuracy at low missingness levels, OTrecod and missMDA show superior robustness in high missingness scenarios. Furthermore, OTrecod excels under severe non-linear constraints. These findings are further supported by subsets of the National Child Development Study, in which OTrecod produced the most stable and consistent recoding alignments across methods.

**Availability and implementation:**

The source code supporting this study is publicly available at https://github.com/FloAI/CompareOT and archived on Zenodo (DOI: 10.5281/zenodo.20542318).

## 1 Introduction

Today, open science is becoming increasingly prevalent in the production and dissemination of scientific knowledge. This approach is based on the transparent sharing of data, methods, and results throughout the research process. Researchers are thus encouraged to make their data openly available to the scientific community. Finding data is thus becoming easier. However, finding data alone is insufficient, and in this context, the Findability, Accessibility, Interoperability, and Reuse (FAIR) principles provide guidelines to go beyond simply finding data and extend to effective reuse and integration. To make these guidelines efficient and robust, methodological developments are essential. In particular, data interoperability often involves integrating heterogeneous databases, originally collected for different purposes. Our contribution is situated within this broader challenge. More specifically, improving database merging, a key step in enabling interoperable and reusable scientific data, is the motivation for our work.

Different techniques are already used to combine heterogeneous databases from diverse sources, leveraging both statistical and symbolic approaches. These include probabilistic models and probabilistic databases through principled probabilistic reasoning (Suciu 2020), and time-dependent or sequential data through Hidden Markov Models and temporal models that align, smooth, and infer latent states across data streams, thereby supporting robust temporal integration (Castanedo 2013). At a higher semantic level, other techniques are used, such as logical reasoning techniques, ontology-based integration, rule engines, and schema mapping, which help reconcile semantic heterogeneity and ensure interoperability between differing data representations (Borowicc and Souza 2024, Putrama and Martinek 2024). More recently, knowledge graph frameworks have emerged for improved scalability and interpretability. Further extensions of these paradigms through hybrid AI approaches combine large language models and graph learning with probabilistic and logical reasoning to automate schema discovery, data fusion, and knowledge validation across heterogeneous systems (Mohamed *et al.* 2025).

One of the main challenges in database integration is variable recoding. This problem emerges especially when a categorical variable of interest is represented using different coding schemes across two different databases. Such inconsistencies often stem from differences in data collection or interpretation methods, leading to significant difficulties in the integration process. We consider Database A, which contains covariates and one version of the target variable $Y_{A}$. Database B contains the same covariates and an alternative encoding of the target variable *Y*, denoted $Y_{B}$. The goal is to create a synthetic database for $Y_{B}$ in database A and $Y_{A}$ in database B (Gares *et al.* 2019). Variable recoding can be formalized as a missing data problem (Gares *et al.* 2019). In this context, missingness depends solely on the database to which a subject belongs rather than the variable itself. To address this, the extensive literature on missing data offers several paradigms, including statistical imputation (Rubin 1987), supervised learning (Jang *et al.* 2020, Alwateer *et al.* 2024), and optimal transport strategies that exploit the transportation plan between the distributions of $Y_{A}$ and $Y_{B}$ (Gares *et al.* 2019, Garès and Omer 2022).

To thoroughly benchmark these established methods, we selected five distinct, state-of-the-art imputation paradigms. The OTrecod algorithm, as the optimal transport approach, Multiple Imputation by Chained Equations (MICE) (van Buuren and Groothuis-Oudshoorn 2011) was chosen as the standard parametric approach. To represent multivariate data analysis, we utilised principal component-based imputation methods (Josse and Husson 2016). Finally, for non-parametric machine learning baselines, we included k-Nearest Neighbours (kNN) (Cover and Hart 1967) for distance-based imputation, and Random Forests (missForest) for tree-based algorithmic imputation (Stekhoven and Bühlmann 2012).

The aim of this paper is to evaluate the performance of the five aforementioned approaches for variable recoding in data merging. Specifically, we explore the effects of different types of data bias on methodological performance. Potential sources of bias include the amount and mechanism of missing data in covariates, correlations between covariates and outcomes, non-linear relationships, and distributional shifts in the variable of interest.

Missing data among the covariates are among the most critical factors when addressing such recoding problems. It poses a fundamental challenge in statistical analysis and machine learning, as it can introduce bias, reduce statistical power, and compromise the validity of conclusions if not properly addressed (Rubin 1987, Little 2024). Such issues occur pervasively across multiple domains, including healthcare, finance, and the social sciences, where incomplete databases often result from measurement errors, data collection constraints, or participant nonresponse (Schafer 1997, van Buuren 2018). To mitigate this, imputation techniques such as MICE (van Buuren and Groothuis-Oudshoorn 2011) are widely used. In this paper, a comprehensive simulation study is conducted to evaluate the effects of data biases arising from correlation, distributional shifts, and missing data.

Background on the different approaches used for the recoding problem and missing data mechanisms is provided in Section 2. Section 3 details the simulation study and the data-generation process, introducing the scenarios designed to evaluate each approach’s performance. The results of the simulation study are presented and discussed in Section 4. To assess practical applicability, these approaches are also evaluated on a real-world database, the National Child Development Study (NCDS), in Section 5. Finally, Section 6 concludes the paper and discusses future work.

## 2 Background

### 2.1 Methods for recoding variables to merge databases

#### 2.1.1 Algorithm OT based on optimal transportation

The idea behind the OT algorithm is to exploit an optimal transportation plan between the probability distributions of $Y_{A}$ and $Y_{B}$. Optimal Transport (OT) theory, originally introduced by Gaspard Monge in 1781 (Monge 1781), seeks a measurable mapping that reallocates mass from one distribution to another while minimising a prescribed transportation cost. In the continuous setting, under suitable regularity assumptions on the cost function (e.g. strict convexity), the optimal transport plan is characterised by a unique transport map that can often be expressed as the gradient of a convex potential (Brenier’s theorem). In the categorical or discrete setting, the transport plan is formulated as a coupling matrix that solves a linear programming problem, commonly referred to as Hitchcock’s transportation problem; in that case, the solution is generally not unique but can be efficiently computed using dedicated optimisation algorithms.

Given two databases *A* and *B*, where $Y_{A}$ and $Y_{B}$ are respectively observed, one can estimate an empirical optimal transport plan by defining a cost function between individuals based on the covariates that are jointly observed in both datasets. This principle underlies the recoding and data fusion approaches proposed in (Gares *et al.* 2019, Garès and Omer 2022), where regularised optimal transport is used to construct a probabilistic linkage between units from the two samples through a cost reflecting their similarity in the common covariate space. The empirical optimal transport plan gives us the estimated number of individuals recoded in each modality of the variable to be recoded. The second step, assigning an individual to a modality, is performed using k-nearest neighbours, thanks to the introduced cost function.

More broadly, recent methodological and computational advances have established OT as a powerful framework for data analysis and integration. It provides a principled way to align and compare probability distributions arising from heterogeneous sources, with applications ranging from domain adaptation to data fusion and covariate shift correction (Courty *et al.* 2017, Peyré and Cuturi 2019). These developments are accompanied by practical implementations, such as the OTrecod R package, which makes OT-based data fusion approaches readily accessible for applied statistical analysis (Guernec *et al.* 2023).

#### 2.1.2 Multivariate Imputation Chained Equations (MICE)

The Multiple Imputation by Chained Equations (MICE) algorithm represents a major advancement within the Multiple Imputation framework (van Buuren and Groothuis-Oudshoorn 2011). MICE adopts a fully conditional specification strategy. It iteratively imputes each incomplete variable using a regression model conditioned on all other variables. This chained procedure allows for flexible model specification.

Each variable with missing data is modelled conditional on the other variables in the dataset as part of the MICE process, which involves running multiple regression models. This makes it possible to represent each variable according to its distribution. Logistic regression is used to model binary outcomes, while linear regression is used to model continuous outcomes (van Buuren and Groothuis-Oudshoorn 2011).

There are four primary steps in the MICE process. Initially, basic placeholder values, such as the mean or mode, are used to impute missing data for each variable. After that, all other values are retained, but the imputed values for one variable are returned to missing. This variable is then treated as the dependent variable, and the other variables are treated as independent predictors when regressed on it. The missing values for that variable are replaced with the produced predictions, often with additional random variation. The imputations can be improved one step at a time until they stabilise by repeating these procedures for every variable with missing data and cycling through several iterations. After that, a number of finished datasets are produced to capture the uncertainty of the imputation process.

MICE is widely used in applied research across the social sciences, psychology, and epidemiology for its practical performance and methodological flexibility. Multivariate relationships have been well established through simulation and empirical research, and they preserve correlations among variables without requiring explicit distributional assumptions.

It is important to distinguish two separate roles played by MICE in this study. First, MICE is used as a recoding method, that is, as one of the algorithms being compared. It is applied directly to predict the missing values of $Y_{A}$ and $Y_{B}$. Secondly, in one of our simulation scenarios (see subsection 3.4), MICE is used as a preprocessing step to impute missing covariate values prior to any recoding.

#### 2.1.3 k-Nearest Neighbours (kNN)

The K-Nearest Neighbours (kNN) algorithm is a non-parametric, instance-based learning method commonly used for both classification and regression tasks (Cover and Hart 1967). The core principle of kNN is to predict the value or class of a given instance. The algorithm identifies the *k* most similar instances, or neighbours, in the feature space and bases its prediction on their values. In classification problems, kNN typically employs majority voting, while in regression, it uses the mean or median of the neighbours’ values. The similarity between instances is generally quantified through distance metrics such as the Euclidean or Manhattan distance.

kNN is also used to solve problems in missing data imputation. Here, kNN relies on the assumption that similar samples possess similar feature values. In this case, the algorithm first identifies one or more missing attributes and their *k* nearest neighbours based on the observed non-missing features. The distances between its variables can be computed, typically using a normalised Euclidean distance, to ensure that all features contribute equally. For continuous variables, the imputed value is often the mean or median of the neighbours’ values, whereas for categorical variables, the most frequent category is used.

#### 2.1.4 missForest

missForest (Stekhoven and Bühlmann 2012) is a non-parametric, iterative imputation method based on the Random Forest algorithm. Unlike traditional single-pass imputation techniques, missForest effectively handles mixed-type datasets comprising both continuous and categorical variables by training a Random Forest on the observed values of a target variable, using the remaining variables as predictors. The algorithm iteratively updates the missing values until a stopping criterion, typically based on the stabilisation of the imputed matrix, is met. By leveraging the inherent strengths of Random Forests, such as the random sampling of features for node splitting and robustness to complex, non-linear interactions, missForest mitigates the risk of overfitting and demonstrates superior robustness to noise. Furthermore, the algorithm utilises out-of-bag (OOB) error estimates to monitor convergence and imputation accuracy without requiring a separate validation set, making it a comprehensive and reliable tool for missing data estimation.

#### 2.1.5 missMDA

missMDA (Josse and Husson 2016) encompasses a family of statistical techniques for handling missing values that leverage Multivariate Data Analysis (MDA) methods, such as Principal Component Analysis (PCA) and Multiple Correspondence Analysis (MCA). Unlike traditional univariate imputation methods, missMDA evaluates the joint distribution and covariance structures across multiple variables, capturing the complex, multidimensional dependencies inherent in the data to uncover latent patterns. A primary objective of missMDA is to estimate missing values by exploiting structural similarities among observations and variables through iterative dimensionality reduction, ensuring that significant structural information is not discarded. By utilising the correlation networks across various predictors, missMDA provides a mathematically rigorous framework for interpreting high-dimensional spaces. This capacity to model simultaneous relationships makes missMDA particularly advantageous for generating stable, data-driven multiple imputations, where the underlying interconnectedness of the variables is leveraged to produce accurate estimates.

### 2.2 Missing data

Various techniques can be employed to deal with missing data, broadly categorised into deletion methods and imputation techniques. Deletion methods, such as list-wise and pairwise deletion, remove incomplete cases from the dataset. Although simple to implement, these approaches can lead to substantial information loss and biased estimates (Rubin 1976, Allison 2002). Basic imputation techniques, such as mean or median imputation, are widely used but can distort variability and underestimate uncertainty (Donders *et al.* 2006). More advanced techniques, including multiple imputation (MI) (Rubin 1987), expectation-maximisation (EM) (Dempster *et al.* 1977), and machine-learning-based imputation (Jang *et al.* 2020, Alwateer *et al.* 2024), offer more robust solutions by leveraging patterns in the data.

According to Rubin (1976) (Little 2024), there are three missingness mechanisms: Missing Completely at Random (MCAR), where the probability of missingness is independent of both observed and unobserved data; Missing at Random (MAR), where missingness depends only on the observed data; and Missing Not at Random (MNAR), where missingness depends on the unobserved data itself.

## 3 Simulation design

**Figure 1:**
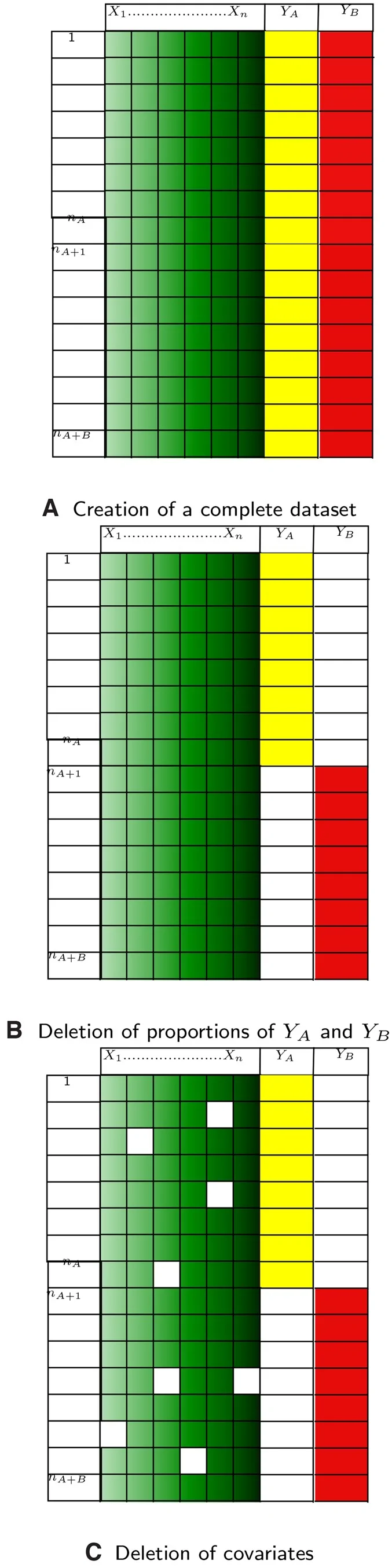
Workflow of dataset generation.

The performance of the different recoding methods under various scenarios is assessed through simulations. Using a set of covariates, we generate a continuous outcome, which we then convert into a categorical one via two discretisations with different numbers of categories, to mimic two databases with different encodings of the same variable *Y*. Then, covariate values are imputed to address missing data. The different steps of the simulation design are illustrated in Fig. 1 and formalised below.

### 3.1 Complete database generation

To generate the complete database, we first consider $\left(X_{1},\ldots ,X_{p}\right)$ to be a set of *p* independent covariates with fixed distributions. Then we construct *Z*, a continuous outcome as a linear combination of the covariates perturbed by an independent Gaussian random noise:


(1) $$Z=\hat{Z}+U\;\text{where}\;\hat{Z}=\beta_{0}+\sum_{j=1}^{p}\beta_{j}X_{j}\;\text{and}\;U∼N(0,\sigma^{2})$$

The strength of association between the covariates and the *Z* may be assessed by means of the coefficient of determination $R^{2}$, which quantifies the proportion of variance in the outcome *Z* that is explained by the covariates and is defined as:


$$R^{2}=\frac{\text{Var}(\hat{Z})}{\text{Var}(Z)}.$$

The distributions of the covariates being given, $\text{Var}(\hat{Z})$, can easily be derived. Noticing that


$$R^{2}=\frac{\text{Var}(\hat{Z})}{\text{Var}(\hat{Z})+\sigma^{2}},$$

and thus


(2) $$\sigma^{2}=\text{Var}(\hat{Z})\frac{1-R^{2}}{R^{2}},$$

It is thus possible to fix the strength of association between the covariates and the outcome by varying the values of $\sigma^{2}$.

To investigate the impact of the assumption of equality of the distribution $Z_{A}$ and $Z_{B}$, two scenarios are considered:

- *Scenarios S (for “same”):* assumes $Z_{A}$ and $Z_{B}$ have the same distribution. To do so, the values of the coefficients in the definitions 1 are the same for $Z_{A}$ and $Z_{B}$.
- *Scenarios D (for “different”):* consider $Z_{A}$ and $Z_{B}$ have not the same distribution. To do so, the values of the coefficients in the definitions 1 are not the same for $Z_{A}$ and $Z_{B}$.

Finally, given $n_{A}$ (resp. $n_{B}$), the sample size of database *A* (resp. *B*), given a value of $R^{2}$, and given a scenario S or D, the complete database generation follows the following steps:

1. Generate $n=n_{A}+n_{B}$ values $\left(x_{1,i},\ldots ,x_{p,i}\right)$, i=1,…,n through their distributions,
2. Generate $n=n_{A}+n_{B}$ values $u_{i}$, i=1,…,n from a centred Gaussian distribution of variance $\sigma^{2}$ given by relation (2).
3. For each i=1,…,n, compute $z_{A,i}$ and $z_{B,i}$ by means of relation (1).
4.
$\left(y_{A,i},i=1,\ldots ,n\right)$ is $\left(z_{A,i},i=1,\ldots ,n\right)$ categorised by quartiles and $\left(y_{B,i},i=1,\ldots ,n\right)$ is $\left(z_{B,i},i=1,\ldots ,n\right)$ categorised by tertiles.

Data generated at this step is illustrated by Sub-Fig. 1a. The data used to test the performances of the algorithms (Sub-Fig. 1b) consists in Database A : $\left(x_{1,i},\ldots ,x_{p,i},y_{A,i},i=1,\ldots ,n_{A}\right)$, and Database B : $\left(x_{1,i},\ldots ,x_{p,i},y_{B,i},i=n_{A}+1,\ldots ,n_{A}+n_{B}\right)$.

Remark 1. The remaining values $\left(y_{A,i},i=n_{A}+1,\ldots ,n_{A}+n_{B}\right)$ and $\left(y_{B,i},i=1,\ldots ,n_{A}\right)$ are used to assess the performance of the algorithm (see Section 3.5).

In order to simulate realistic patterns resembling those observed in the National Child Development Study (NCDS) (see Subsection 5 for details), five covariates are considered with the distributions listed in Table 1.

**Table 1:** Distributions of the covariates for the simulation study.

Variable	Distribution	Parameters
$X_{1}$	Bernoulli $b(\pi_{1})$	$\pi_{1}=0.5$
$X_{2}$	Multinomial $M(\pi_{21},\pi_{22},\pi_{23})$	$\pi_{21}=0.3,\;\pi_{22}=0.4,$ $\pi_{23}=0.3$
$X_{3}$	Multinomial $M(\pi_{31},\ldots ,\pi_{34})$	$\pi_{31}=0.1,\;\pi_{32}=0.2,$ $\pi_{33}=0.3,\;\pi_{34}=0.4$
$X_{4}$	Bernoulli $b(\pi_{4})$	$\pi_{4}=0.5$
$X_{5}$	Uniform U(a,b)	a=20, b=80

For scenario S, the coefficients are fixed to:


$$\beta_{0}=20,\;\beta_{1}=5,\;\beta_{2}=10,\;\beta_{3}=2,\;\beta_{4}=-8,\;\beta_{5}=0.3.\;$$

And for scenario D, they are fixed to


$$\beta_{0}^{\left(A\right)}=20,\;\beta_{1}^{\left(A\right)}=5,\;\beta_{2}^{\left(A\right)}=10,\;\beta_{3}^{\left(A\right)}=2,\;\beta_{4}^{\left(A\right)}=-8,\;\beta_{5}^{\left(A\right)}=0.3,$$

for $Z_{A}$ and to


$$\beta_{0}^{\left(B\right)}=15,\;\beta_{1}^{\left(B\right)}=4,\;\beta_{2}^{\left(B\right)}=8,\;\beta_{3}^{\left(B\right)}=1.5,\;\beta_{4}^{\left(B\right)}=-6,\;\beta_{5}^{\left(B\right)}=0.25,\;$$

for $Z_{B}$.

#### 3.1.1 Alternative functional forms for the latent outcome

While the baseline specification in Equation (1) assumes a linear relationship between the covariates and the latent outcome, we also consider several alternative functional forms to assess the robustness of the recoding methods under collinearity and non-linearity.

*(i) Sigmoid transformation*

To model a bounded non-linear response, we apply a sigmoid transformation to a linear predictor:


(3) $${\hat{Z}}_{L}=5X_{1}+10X_{2}+2X_{3}-8X_{4}+0.3X_{5}-29.5$$

and define the latent signal as:


(4) $${\hat{Z}}_{\text{sigm}}=\frac{100}{1+e^{-{\hat{Z}}_{L}}}$$

*(ii) Exponential transformation*

To investigate the impact of scale and skewness, we consider an exponential transformation of the linear predictor:


(5) $${\hat{Z}}_{\;\text{exp}\;}=\text{exp}({\hat{Z}}_{L})$$

*(iii) Collinearity*

In the baseline setting, $\left(X_{1},\ldots ,X_{p}\right)$ are generated as independent covariates with fixed marginal distributions. To assess the impact of dependence between predictors, we consider a collinearity scenario in which a subset of the covariates is generated with controlled correlation.

Specifically, the variables $\left(X_{4},X_{5}\right)$ are binary variables jointly generated from a bivariate normal distribution:


(6) $$\begin{array}{c} (X_{4}^{*},X_{5}^{*})∼N\left(\left(\begin{array}{c} 0\\ 0 \end{array}\right),\left(\begin{array}{cc} 1 & \rho \\ \rho & 1 \end{array}\right)\right), \end{array}$$

where ρ∈{0.1,0.5,0.7} controls the strength of dependence. The observed covariates are then obtained via marginal transformations: $X_{4}$ is defined as a binary variable by thresholding $X_{4}^{*}$ at zero, and $X_{5}$ is a uniformly distributed variable and obtained by applying the standard normal cumulative distribution function to $X_{5}^{*}$.

All remaining covariates are generated independently according to their respective marginal distributions.

### 3.2 Introduction of missing data in the covariates

In order to investigate the behaviour of the algorithm when data is missing in the covariates, a given proportion *P* of values $\left((x_{1,i},\ldots ,x_{p,i}\right)$, i=1,…,n) are randomly deleted according to the missing data mechanism chosen. The resulting datasets *A* and *B* are illustrated by Sub-Fig. 1c. To generate missing data for the covariates (amputation procedures), the R-miss-tastic package developed by Mayer *et al.* (2022) is used. R-miss-tastic is a platform for data amputation based on (Schouten *et al.* 2018). The authors propose a multivariate imputation procedure that accurately simulates missingness under various mechanisms (MCAR, MAR, MNAR), with precise control over which variables are affected, how much data is missing, and the pattern of missingness. This method enables the construction of missing data scenarios that closely reflect real-world conditions.

### 3.3 Missingness management

To deal with the issue of missing data management, three strategies may be considered:

- *Complete Case (CC):* Here no missing data is introduced. The covariates are complete and remain so.
- *Incomplete Case (IncC):* Also, missing data is introduced following one of Rubin’s missingness mechanisms at a time. The objective is to examine how covariate missingness affects the performance of recoding methods across different target variables and distributional shifts.
- *Imputed Case (ImpC):* In this case, missing data in the covariates is imputed prior to recoding using MICE. MICE is chosen to estimate plausible values of the missing entries before variable recoding and analysis.

### 3.4 Simulation scenarios

The simulation scenarios aim to build databases A and B in order to investigate the performance of the five reconstruction algorithms regarding

1. Assumption on the distributions (AonD) of $Y_{A}$ and $Y_{B}$, where *S* denotes identical distributions and *D* denotes different distributions.
2. Functional form of the latent outcome, defined by the specification of *Z*, namely $Z_{L}$, $Z_{\;\text{exp}\;}$, and $Z_{\text{sigm}}$.
3. Sample size, denoted by $n=n_{A}+n_{B}$.
4. Ratio of observations between Database A and Database B, defined as $F=n_{A}/n_{B}$.
5. Strength of association between the covariates and the outcome, controlled by $R^{2}$.
6. Degree of collinearity between covariates, controlled by ρ.
7. Number of modalities, denoted by *m*, controlling the granularity of the categorical outcomes.
8. Missing data management strategy (MMD), together with the proportion of missingness in the covariates, denoted by *P*.
9. Missing data mechanism (MDM), taking values MCAR, MAR, or MNAR.

Table 2 fixes the values of the parameters of the different scenarios that were considered.

**Table 2:** Parameters of the simulation scenarios.

Scen.	MMD	Func.	ρ	Modalities	AonD	*n*	*F*	$R^{2}$	*P*	MDM
1	CC	$Z_{L}$	–	(4,3)	S	500	{0.1, 1, 9}	0.9	**–**	**–**
2	CC	$Z_{L}$	–	(4,3)	S	{100, …, 1000}	1	0.9	–	–
3	CC	$Z_{L}$	–	(4,3)	S	500	1	{0.2, 0.5, 0.9}	–	–
4	CC	$Z_{L}$	{0.1, 0.5, 0.9}	(4,3)	S	1000	1	0.9	–	–
5	CC	$Z_{\;\text{exp}\;}$	–	(4,3)	S	{100, …, 1000}	1	0.9	–	–
6	CC	$Z_{\text{sigm}}$	–	(4,3)	S	{100, …, 1000}	1	0.9	–	–
7	CC	$Z_{L}$	–	(4,3)	D	{100, …, 1000}	1	0.8	–	–
8	IncC	$Z_{L}$	–	(4,3)	S	{100, …, 1000}	1	1	{0.1, 0.5, 0.9}	MCAR
9	IncC	$Z_{L}$	–	(4,3)	S	{100, …, 1000}	1	1	{0.1, 0.5, 0.9}	MAR
10	IncC	$Z_{L}$	–	(4,3)	S	{100, …, 1000}	1	1	{0.1, 0.5, 0.9}	MNAR
11	ImpC	$Z_{L}$	–	(4,3)	S	{100, …, 1000}	1	1	{0.1, 0.5, 0.9}	MCAR
12	ImpC	$Z_{L}$	–	(4,3)	S	{100, …, 1000}	1	1	{0.1, 0.5, 0.9}	MAR
13	ImpC	$Z_{L}$	–	(4,3)	S	{100, …, 1000}	1	1	{0.1, 0.5, 0.9}	MNAR

MMD, Management of missing data; AonD, assumption on distribution; MDM, Missing Data Mechanism; ρ, Correlation.

Remark 2. The details on the tuning of algorithm parameters are found in Section 1 of the Supplementary Materials, available as supplementary data at *Bioinformatics* online.

### 3.5 Performance of recoding algorithms

Consider Databases A and B, generated according to the procedure described in the previous section. When present, missing data are handled using one of the strategies introduced in Section 3.3: CC, IncC, or ImpC. After this step, the resulting dataset—either reduced by removing individuals or completed via imputation—corresponds to Sub-Fig. 1b. Recoding can then be performed using OTrecod, MICE, kNN, missForest, or missMDA. To quantify variability, results are averaged over 30 simulation runs, and the corresponding standard deviations are reported.

These algorithms produce imputed outcome values denoted $\left(y_{A\to B,i},i=n_{A}+1,\ldots ,n_{A}+n_{B}\right)$ for Database B and $\left(y_{B\to A,i},i=1,\ldots ,n_{A}\right)$ for Database A.

#### 3.5.1 Precision

The performance, for a given scenario, missing data handling strategy, and recoding algorithm, is assessed by:


(7) $$\mathrm{Perf}=\frac{1}{n_{A}}\sum_{i=1}^{n_{A}}I(y_{A\to B,i}=y_{A,i})+\frac{1}{n_{B}}\sum_{i=1}^{n_{B}}I(y_{B\to A,i}=y_{B,i})$$

#### 3.5.2 Cohen’s Kappa

To account for agreement occurring by chance, particularly in the presence of class imbalance, we compute Cohen’s Kappa separately for each recoding direction and then aggregate the results.

Let $\kappa_{A}$ (resp. $\kappa_{B}$) denote the Cohen’s Kappa computed between $\left(y_{B\to A,i},i=1,\ldots ,n_{A}\right)$ and $\left(y_{B,i},i=1,\ldots ,n_{A}\right)$ (resp. between $\left(y_{A\to B,i},i=1,\ldots ,n_{B}\right)$ and $\left(y_{A,i},i=1,\ldots ,n_{B}\right)$). Each coefficient is defined as:


(8) $$\kappa_{A}=\frac{p_{o}^{\left(A\right)}-p_{e}^{\left(A\right)}}{1-p_{e}^{\left(A\right)}},\;\kappa_{B}=\frac{p_{o}^{\left(B\right)}-p_{e}^{\left(B\right)}}{1-p_{e}^{\left(B\right)}}$$

where $p_{o}^{\left(A\right)}$ and $p_{o}^{\left(B\right)}$ denote the observed agreement, and $p_{e}^{\left(A\right)}$ and $p_{e}^{\left(B\right)}$ the expected agreement under independence, computed from the marginal distributions of the true and recoded categories in each database.

#### 3.5.3 Mean absolute error (MAE)

Since the target variables are ordinal, categorical levels are mapped to consecutive integers. This allows us to quantify the magnitude of recoding errors, penalising predictions that are further from the true category.

For a given scenario, missing data handling strategy, and recoding algorithm, the Mean Absolute Error is defined as:


(9) $$\mathrm{MAE}=\frac{1}{n_{A}}\sum_{i=1}^{n_{A}}\left|y_{B\to A,i}-y_{B,i}\right|+\frac{1}{n_{B}}\sum_{i=1}^{n_{B}}\left|y_{A\to B,i}-y_{A,i}\right|$$

where $y_{A\to B,i}$ and $y_{B\to A,i}$ denote the recoded values, and $y_{A,i}$ and $y_{B,i}$ the corresponding true ordinal categories expressed as integers. We can define MAE_Score as


(10) $$\text{MAE}\_\text{Score}=1-\frac{\mathrm{MAE}}{\text{MAE}\_\text{max}}$$

where MAE_max is the greatest value of MAE. Our goal is to obtain an increasing normalised MAE value to facilitate the interpretation of results.

## 4 Results

### 4.1 Complete case (CC)

**Figure 2:**
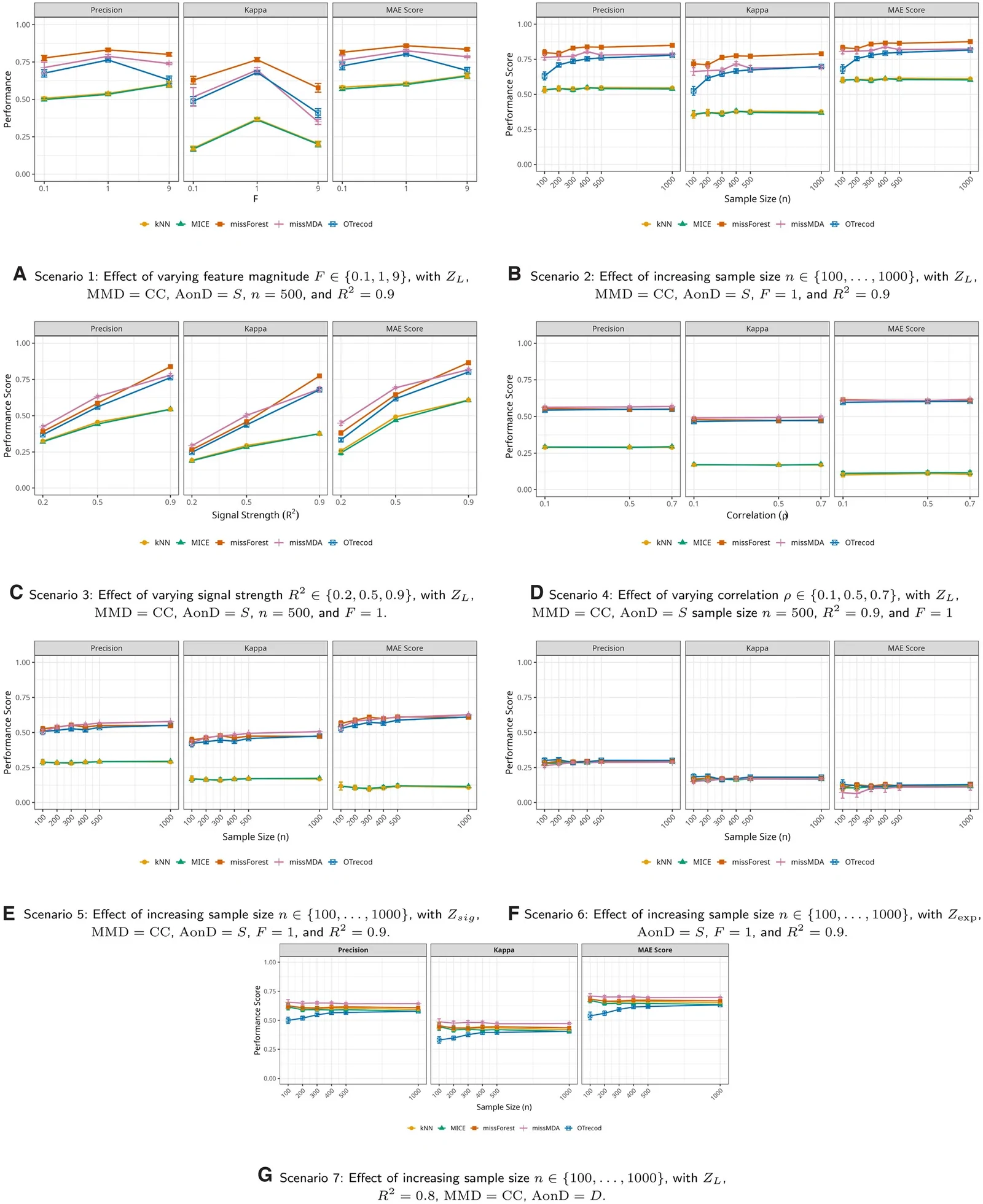
Scenarios 1–7 under complete cases (CC) exploring the effect of varying sample size (*n*), feature magnitude (*F*), signal strength ($R^{2}$), correlation (ρ), and Assumption on the distributions (AonD) mechanisms.

The results in Fig. 2a reveal that when *F* is small (F=0.1), missForest achieves the highest performance across all three metrics (Precision 0.777±0.017, Kappa 0.629±0.026, MAE Score 0.815±0.014), followed closely by missMDA and OTrecod. For the balanced case (F=1), missForest again leads (Precision 0.831±0.010), with missMDA and OTrecod in second and third position, respectively, while MICE and kNN lag behind substantially. When the ratio is reversed (F=9), missForest remains the strongest performer (Precision 0.801±0.011), though the gap between methods narrows. Taken together, the results confirm a V-shaped pattern for some methods as *F* goes away from balance.

Figure 2b shows the effect of the total sample size *n* increasing from 100 to 1000 on performance. As *n* grows, missForest consistently leads, reaching precision 0.849±0.005 and MAE Score 0.875±0.004 at n=1000. OTrecod also improves substantially with sample size and approaches missForest at larger *n*, while missMDA remains competitive throughout. MICE and kNN exhibit comparatively modest gains, indicating limited scalability. Their precision scores plateau around 0.54–0.55 regardless of *n*, suggesting they are less capable of exploiting additional data to refine their results.

Figure 2c shows results as the coefficient of determination $R^{2}$ increases from 0.2 to 0.9. At low signal strength ($R^{2}=0.2$), missMDA achieves the highest precision (0.423±0.010) and MAE Score (0.448±0.015), outperforming all other methods, including missForest and OTrecod. As $R^{2}$ increases to 0.9, missForest takes the lead (precision 0.838±0.008, MAE Score 0.866±0.007), while missMDA and OTrecod follow. MICE and kNN improve with $R^{2}$ but remain substantially below the top-performing methods at all levels.

Interestingly, the results in Fig. 2d reveal that the degree of correlation between variables (ρ) has no notable impact on recoding performance. For instance, as ρ increases from 0.1 to 0.7, the performance metrics of the top methods remain remarkably stable. missMDA Precision shifts negligibly from 0.563±0.009 to 0.568±0.008, and OTrecod Kappa remains constant at ∼0.472.

Figures 2e and f illustrate the effect of introducing non-linear associations between the covariates and the outcome. Under a sigmoid relationship (Fig. 2e), missForest effectively captures the non-linear structure, yielding the highest precision and MAE scores across most sample sizes at n=300 (Precision 0.553±0.009, MAE Score 0.610±0.008), with missMDA also performing strongly as *n* increases. However, in the case of an exponential relationship, as can be seen in Fig. 2f, the performance of all standard machine learning methods collapses significantly, with Kappa scores plunging to the 0.15–0.17 range even at n=1000. Under this extreme constraint, OTrecod emerges as the most resilient performer across all sample sizes, maintaining the highest metrics at n=1000 (Precision 0.301±0.006, Kappa 0.182±0.007).

Figure 2g presents the effect of increasing sample size under a distributional shift scenario (AonD=D), where the underlying data-generating coefficients differ between databases A and B. Under these mismatched conditions, missMDA consistently outperforms all other methods across every sample size and metric, achieving the highest precision, Kappa, and MAE scores at n=1000 (Precision 0.644±0.006, Kappa 0.473±0.008). Interestingly, however, OTrecod is the only method that exhibits a distinct, monotonic improvement as sample size increases, precision grows from 0.500 at n=100 to 0.578 at n=1000.

### 4.2 Incomplete case (IncC)

**Figure 3:**
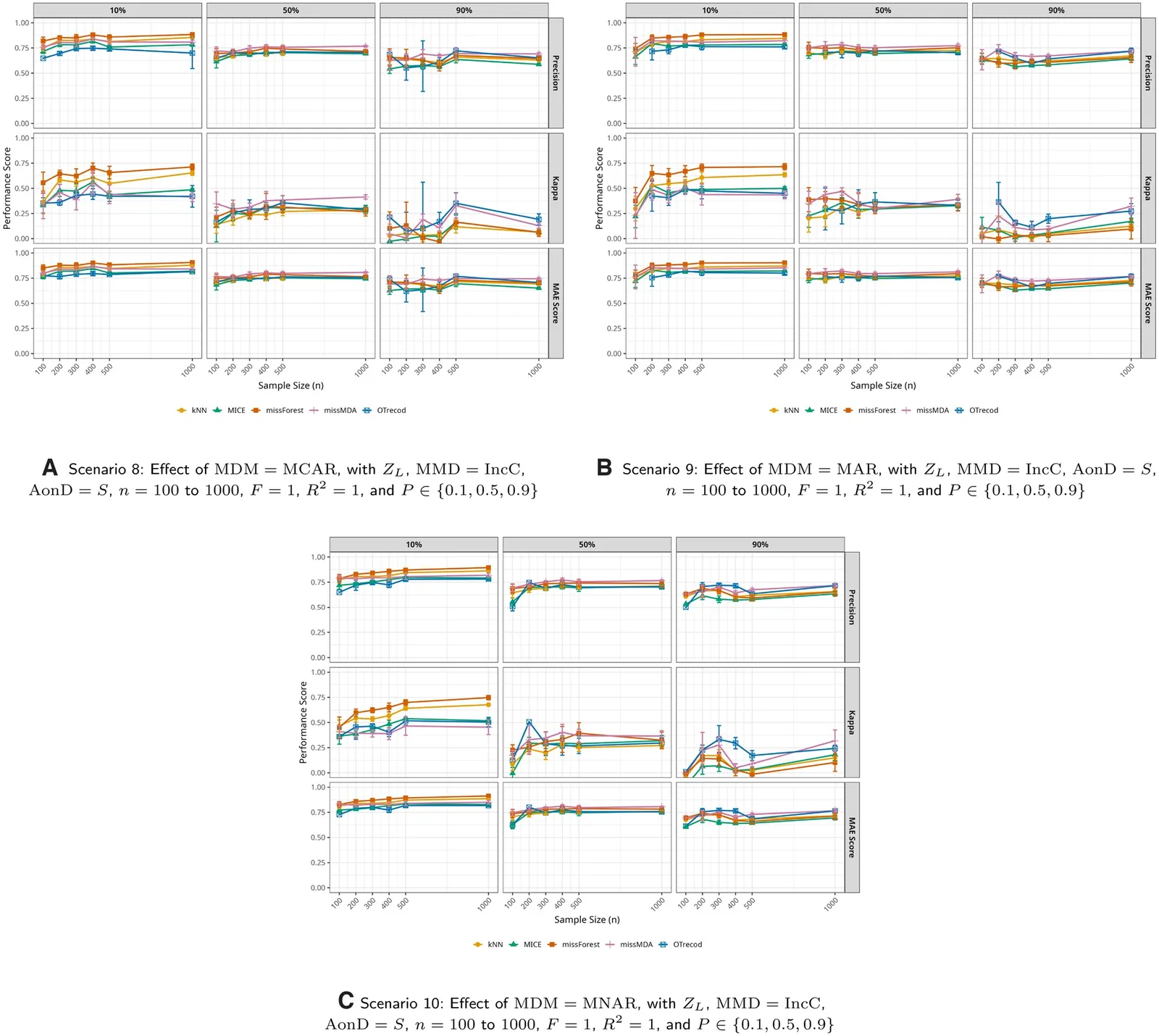
Scenarios 8–10 involve incomplete cases (IncC) under different MDM (MCAR, MAR, MNAR) with fixed *F*, $R^{2}$, and varying proportions of missingness *P*.

The results in Fig. 3 show that at low missingness (P=0.1) when missing values in covariates are retained without imputation, missForest achieves the highest performance across all mechanisms, under MCAR for n=1000 (Precision 0.884±0.014, Kappa 0.713±0.029, MAE Score 0.906±0.010), followed by missMDA and kNN. When the missingness increases to P=0.5, missMDA becomes the strongest performer (Precision 0.767±0.009), with OTrecod and missForest trailing closely behind. Under extreme missingness (P=0.9), OTrecod and missMDA emerge as the most robust methods; for instance, missMDA maintains a Precision of 0.691±0.014, and OTrecod achieves 0.651±0.025, while MICE and kNN degrade severely.

### 4.3 Imputed case (ImpC)

**Figure 4:**
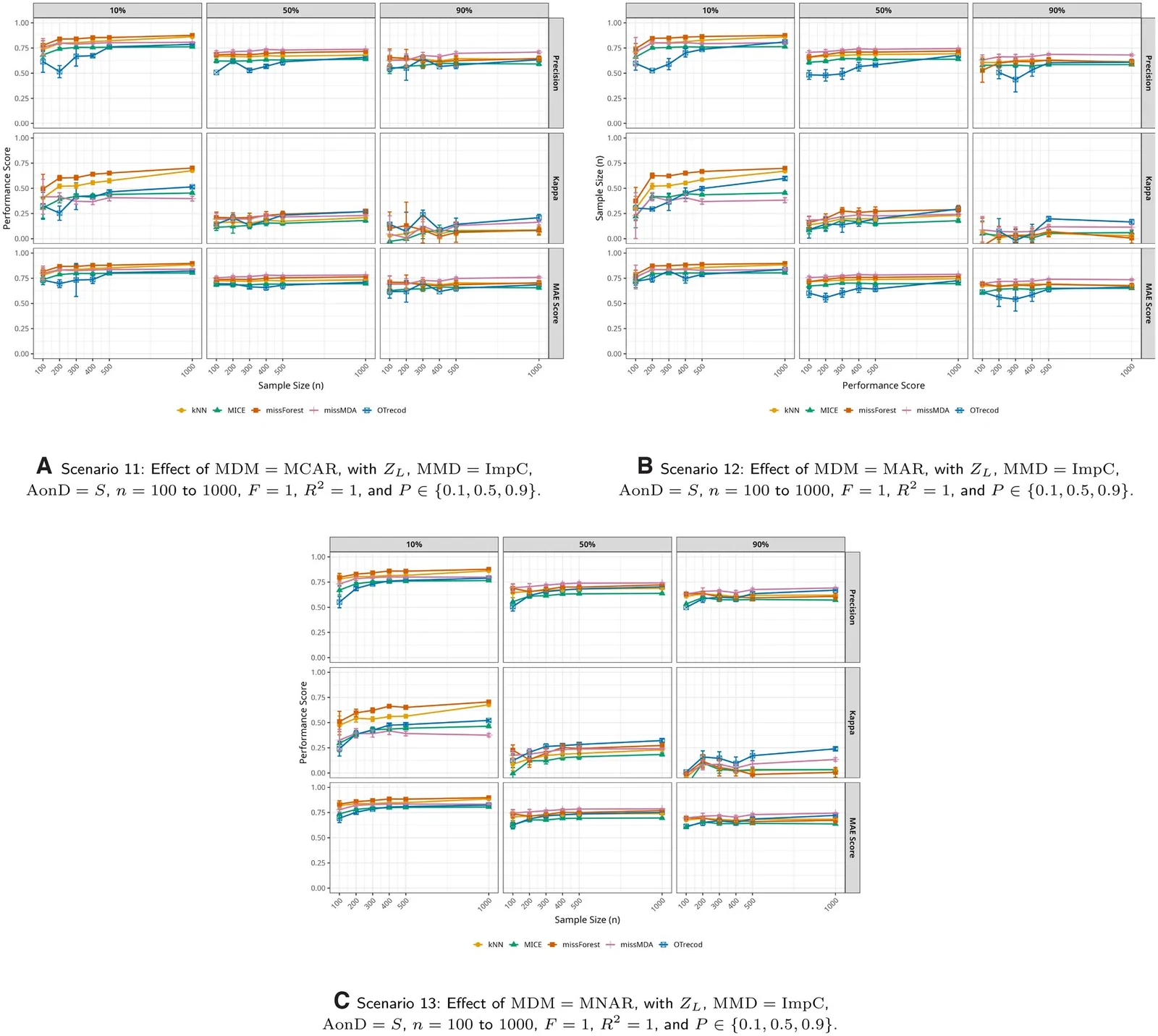
Scenarios 11–13 involve imputed cases (ImpC) under different MDM (MCAR, MAR, MNAR) with fixed *F*, $R^{2}$, and varying proportions of missingness *P*.

The results in Fig. 4 show the performance after imputing missing covariate values prior to recoding. At low missingness (P=0.1), missForest again leads across mechanisms, under MCAR for n=1000 (Precision 0.876±0.004, Kappa 0.702±0.009, MAE Score 0.897±0.004), followed closely by kNN and missMDA. At moderate missingness (P=0.5), missMDA asserts its position as the best-performing method overall (Precision: 0.737±0.004), yielding more stable results than in the incomplete-case setting. Under high missingness (P=0.9), missMDA maintains its lead (Precision 0.709±0.013), while OTrecod recovers relative to the other methods, with MICE and kNN remaining the weakest performers.

## 5 Experimentation on real dataset

In real data, we do not have the true outcome; therefore, we cannot compare performance assessments. We are therefore interested in the agreement among the results obtained by the five approaches across three criteria: concordance to assess similarity of results between methods, Wasserstein distance to assess pairwise agreement in terms of distributions, and, at the individual level, ROC AUC to assess downstream classification performance.

### 5.1 Description of the NCDS dataset

The experiment is conducted using data from the National Child Development Study (NCDS), a long-term longitudinal survey that has followed the lives of over 17 000 individuals born in a single week in 1958 across England, Scotland, and Wales. The NCDS collects extensive information on multiple life domains, including physical and educational development, socioeconomic circumstances, employment trajectories, family composition, health status, well-being, social participation, and attitudes. Among the numerous variables recorded, particular attention is given to the participants’ social class, measured using two occupation-based classification systems collected at each wave of data collection.

The first of these was the Goldthorp Social Class ’90 scale (GSS90), an ordered scale with 11 categories, while the second was the RGs Social Class ’91 scale (RGS91), an ordered scale with only 6 categories. The social class of subjects was treated as the outcome and was intended to be measured only with the GSS90 scale in the first dataset generated (DB1), whereas it was assumed to be measured only with the RGS91 scale in the second dataset (DB2).

The aim of the study was to resolve the recoding problem in each of the two datasets to obtain a unique scale across both bases. DB1 contains 4 covariates and 5476 observations, while DB2 contains 4 covariates and 365 observations.

### 5.2 Evaluation metrics for NCDS dataset

*(i) Concordance:*

Agreement between the categorical predictions is quantified by the concordance rate (the proportion of agreement). For each method *m*, concordance is computed for $Y_{A}$ and $Y_{B}$ relative to the recoding method and reported separately:


(11) $$C_{A}^{\left(m\right)}=\text{Conc}({\hat{Y}}_{A}^{\left(m_{1}\right)},{\hat{Y}}_{A}^{\left(m_{2}\right)}),\;C_{B}^{\left(m\right)}=\text{Conc}({\hat{Y}}_{B}^{\left(m_{1}\right)},{\hat{Y}}_{B}^{\left(m_{2}\right)}).$$

In this discrete categorical setting, the concordance between two vectors of predictions *p* and *q* of length *n* is computed as:


(12) $$\text{Conc}(p,q)=\frac{1}{n}\sum_{i=1}^{n}I(p_{i}=q_{i}),$$

where I(·) is the indicator function, yielding 1 if the predicted categories match exactly and 0 otherwise.

*(ii) Wasserstein Distance:*

Distributional discrepancies are quantified using the first-order Wasserstein distance. For each method *m*, distances are computed for $Y_{A}$ and $Y_{B}$ relative to the recoding method and reported separately:


(13) $$W_{A}^{\left(m\right)}=W_{1}(L({\hat{Y}}_{A}^{\left(m_{1}\right)}),L({\hat{Y}}_{A}^{\left(m_{2}\right)})),\;W_{B}^{\left(m\right)}=W_{1}(L({\hat{Y}}_{B}^{\left(m_{1}\right)}),L({\hat{Y}}_{B}^{\left(m_{2}\right)})).$$

In the discrete setting, $W_{1}$ is computed as:


(14) $$W_{1}(p,q)=\sum_{k}\left|F_{p}(k)-F_{q}(k)\right|.$$

*(iii) ROC AUC:*

Discriminative performance is assessed using the Area Under the Receiver Operating Characteristic Curve (ROC AUC). For each method, AUC is computed separately for both recoding directions and averaged:


(15) $$\mathrm{AUC}=\frac{1}{2}\left({\mathrm{AUC}}_{A}+{\mathrm{AUC}}_{B}\right),$$

where ${\mathrm{AUC}}_{A}$ (resp. ${\mathrm{AUC}}_{B}$) is computed from $\left(y_{B\to A,i},y_{B,i}\right)$ for $i=1,\ldots ,n_{A}$ (resp. $\left(y_{A\to B,i},y_{A,i}\right)$ for $i=1,\ldots ,n_{B}$). The AUC is defined as


(16) $$\mathrm{AUC}=\int_{0}^{1}\text{TPR}(t)d(\text{FPR}(t)).$$

where TPR is the true positive rate, and FPR is the false positive rate.

Because the true outcome is unavailable across both original databases, direct evaluation of recoding accuracy is not possible. Therefore, to assess discriminative performance (Accuracy and ROC AUC), we utilized a downstream classification task. For each completed dataset produced by a recoding method, we trained a Random Forest classifier on a 70% training split to predict the target variable (using the covariates as predictors). Performance was then evaluated on the remaining 30% hold-out test set. Because the target variables GSS90 and RGS91 are ordinal and multi-categorical, the standard binary ROC AUC formulation is implemented via the pROC package in R (Robin *et al.* 2011). This approach computes the ROC AUC for all possible pairwise class combinations (one-vs-one) and averages them to yield a single comprehensive multiclass AUC metric.

### 5.3 Results

We consider cases similar to those used in the simulations described in Section 3 above.

- CC: We remove the entries with missing covariates in the dataset. Only observations with fully recorded values for all covariates and the social class variable were retained for analysis. $Y_{A}$ contains 307 observations while $Y_{B}$, 4013 observations.
- IncC: We keep the missing covariates in the original datasets, we do not impute them, and we run the methods on this. A total of 5035 observations for 6 variables are recorded, of which $Y_{A}$ has 365 observations and $Y_{B}$ has 4670 observations.
- ImpC: We impute missing entries in the covariates and maintain the original size of the dataset. A total of 5035 observations for 6 variables are recorded, of which $Y_{A}$ has 365 observations and $Y_{B}$ has 4670 observations.

#### 5.3.1 Concordance

Table 3 reports pairwise concordance percentages across the three handling strategies (CC, IncC, ImpC) for both $Y_{A}$ and $Y_{B}$. It can be noted that OTrecod and missForest are by far the most concordant pairs, agreeing on 63% of observations for $Y_{A}$ and 59% for $Y_{B}$ in the complete case, with similarly high agreement under IncC and ImpC.

**Table 3:** Concordance percentages among OTrecod, kNN, MICE, missForest (MF), and missMDA (MDA) for $Y_{A}$ and $Y_{B}$.

	CC		IncC		ImpC	
Comparison	$Y_{A}$	$Y_{B}$	$Y_{A}$	$Y_{B}$	$Y_{A}$	$Y_{B}$
OT-kNN	12.38	22.43	12.88	22.21	13.15	22.61
OT-MICE	29.32	37.20	28.77	35.61	27.67	34.56
OT-MF	**63.19**	**58.51**	**49.59**	**54.69**	**64.11**	**66.27**
OT-MDA	0.33	0.00	0.27	0.00	0.27	0.00
kNN-MICE	11.07	21.08	12.60	21.69	15.62	22.48
kNN-MF	14.33	25.07	16.99	24.30	16.71	24.11
kNN-MDA	1.30	0.00	2.47	0.00	2.47	0.00
MICE-MF	23.78	38.77	27.40	44.73	24.93	37.69
MICE-MDA	1.63	0.00	1.10	0.00	4.38	0.00
MF-MDA	0.00	0.00	0.00	0.00	0.00	0.00
All 5	0.00	0.00	0.00	0.00	0.00	0.00
*n*	307	4013	365	4670	365	4670

The highest pairwise agreements per column are highlighted in bold.

OTrecod and MICE also show moderate pairwise agreement 27%–29% for $Y_{A}$ and 35%–37% for $Y_{B}$, while kNN-based pairs show lower concordance with all other methods. In sharp contrast, OTrecod and missMDA agree on fewer than 1% of observations for $Y_{A}$ and essentially 0% for $Y_{B}$ across all three scenarios, Consequently, no observations are recoded identically by all five methods.

#### 5.3.2 Wasserstein distances

In Table 4, the discrete first-order Wasserstein distances between the predicted outcome distributions of each method pair are used to understand the recoding behaviour of the different methods in the absence of ground truth. OTrecod and MICE yield the most similar recoded outcome distributions, with distances below 0.035 for $Y_{A}$ and below 0.105 for $Y_{B}$ across all scenarios.

**Table 4:** Discrete 1D Wasserstein distances among OTrecod, kNN, MICE, missForest (MF), and missMDA (MDA) for $Y_{A}$ and $Y_{B}$.

	CC		IncC		ImpC	
Comparison	$Y_{A}$	$Y_{B}$	$Y_{A}$	$Y_{B}$	$Y_{A}$	$Y_{B}$
OT-kNN	0.074	0.566	0.081	0.459	0.084	0.386
OT-MICE	**0.031**	**0.065**	**0.034**	**0.105**	**0.017**	**0.064**
OT-MF	0.040	0.338	0.061	0.374	0.064	0.320
OT-MDA	3.106	1.303	3.159	1.270	3.154	1.271
kNN-MICE	0.099	0.591	0.109	0.485	0.094	0.390
kNN-MF	0.069	0.528	0.088	0.558	0.057	0.526
kNN-MDA	3.223	1.042	3.263	1.043	3.266	1.040
MICE-MF	0.066	0.336	0.091	0.370	0.069	0.371
MICE-MDA	3.060	1.323	3.113	1.260	3.149	1.273
MF-MDA	3.139	1.226	3.189	1.431	3.262	1.386
*n* missing	307	4013	365	4670	365	4670

Lower values indicate greater distributional alignment, with the best pairwise distances for each column highlighted in bold.

By contrast, all comparisons involving missMDA yield larger Wasserstein distances, exceeding 1.27 for $Y_{B}$ and 3.10 for $Y_{A}$, confirming that missMDA generates a fundamentally different recoded distribution from all other methods. The distances for OTrecod versus kNN and OTrecod versus missForest are intermediate, consistent with the moderate-to-high concordance seen at the individual level.

#### 5.3.3 Discriminative performance

Table 5 reports accuracy and ROC AUC for each method across the three scenarios of CC, IncC and ImpC. OTrecod substantially outperforms all other methods on both targets. For $Y_{A}$, OTrecod achieves a ROC AUC of 0.812 (CC), 0.761 (ImpC), and 0.753 (IncC), compared to at most 0.68 for any other method. For $Y_{B}$, OTrecod reaches a higher ROC AUC of 0.995 (CC) and above 0.977 in all scenarios, while missForest and kNN plateaued around 0.74–0.80. MICE and missMDA, on the other hand, remain below 0.68.

**Table 5:** Comparison of Accuracy and ROC AUC for targets $Y_{A}$ and $Y_{B}$ under CC, impC, and incC scenarios.

Target	Scenario	Metric	OTrecod	MICE	kNN	missForest	missMDA
$Y_{A}$	CC	Accuracy	0.7577	0.4113	0.3426	0.5710	0.4429
		ROC AUC	0.8121	0.6518	0.5944	0.6755	0.6107
	impC	Accuracy	0.7472	0.4044	0.3700	0.5917	0.4626
		ROC AUC	0.7606	0.6560	0.5907	0.6729	0.6135
	incC	Accuracy	0.7505	0.4533	0.3283	0.6142	0.4653
		ROC AUC	0.7533	0.6605	0.5860	0.6769	0.6235
$Y_{B}$	CC	Accuracy	0.9861	0.5031	0.9560	0.9792	0.9552
		ROC AUC	0.9951	0.6746	0.7393	0.7401	0.6707
	impC	Accuracy	0.9808	0.4567	0.9484	0.9801	0.9623
		ROC AUC	0.9775	0.6738	0.7432	0.7961	0.6661
	incC	Accuracy	0.9874	0.4957	0.9471	0.9835	0.9610
		ROC AUC	0.9845	0.6663	0.7455	0.7475	0.6734

The values in bold represent the best values.

Accuracy results follow a similar pattern. OTrecod achieves high accuracy for $Y_{B}$ (0.986 in CC) and the highest accuracy for $Y_{A}$ across all three scenarios. These results confirm that, despite missMDA generating a markedly different recoded distribution and missForest agreeing most often with OTrecod at the individual level, OTrecod exhibits better discriminative performance.

## 6 Discussion and conclusion

Variable recoding is a fundamental challenge in data merging. A critical question for researchers is whether to utilise methods, such as optimal transport, or machine learning pipelines when reconciling disparate datasets. This study investigated the performance of five distinct algorithms, OTrecod, MICE, kNN, missForest, and missMDA, under varying structural conditions (sample size *n*, determination coefficient $R^{2}$, distribution shift (AonD = D), correlation ρ, and group size ratio *F*), as well as across differing non-linear relationships and missing data mechanisms.

Across our baseline linear simulation scenarios, the algorithms split into two distinct performance tiers: the group comprising missForest, missMDA, and OTrecod consistently and significantly outperformed MICE and kNN. As the total sample size (*n*) and the signal-to-noise ratio ($R^{2}$) increased, the performance of the recoding methods improved. Interestingly, the degree of correlation between covariates (ρ) demonstrated no notable impact on recoding efficacy. When varying the group size ratio (*F*), the results showed a distinct V-shaped curve as *F* moved away from balance (F=1). This V-shape is a logical consequence of extreme structural imbalance; notably, the effect of *F* was not identical across all indicators.

When evaluating non-linear scenarios of sigmoid and exponential relationships, the algorithms were highly sensitive to the underlying data structure. While missForest efficiently captured sigmoid relationships, introducing an exponential relationship caused a significant collapse in performance across most machine learning methods. In this exponential scenario, missMDA yielded the poorest results, exposing the limitations of its linear assumption foundation. Conversely, OTrecod demonstrated the highest resilience and emerged as the top performer, closely followed by missForest.

When assessing incomplete data scenarios (IncC), the results across missingness mechanisms, MCAR, MAR, and MNAR, remained relatively clustered. This indicates that the proportion of missing data had a much greater impact on the methods’ performance than the specific missingness mechanism. When the percentage of missingness is low, missForest delivers the highest precision across all mechanisms. However, as missingness increases, OTrecod and missMDA emerge as the vastly superior methods.

Crucially, our evaluation of imputed covariate scenarios revealed an important imputation paradox. Generally, imputing missing covariates prior to merging had little positive effect on overall recoding performance. For OTrecod specifically, covariate imputation actively degraded performance. Imputation completes the datasets artificially introducing noise and distorting the true distributional geometry that the optimal transport plan relies upon.

These simulated dynamics were reflected in our application to the real-world NCDS dataset. The choice of recoding strategy did not significantly alter the overall agreement between the top methods. However, OTrecod generated the most consistent and stable recoding alignments, whereas kNN and MICE frequently disagreed. It is important to note that the following practical recommendations are primarily derived from the controlled conditions of our simulation study, while the NCDS analysis serves as a supporting real-world illustration of these dynamics.

This study provides a comprehensive baseline for selecting variable recoding algorithms across different real-life situations with missing covariate data. Based on our empirical findings, we offer the following practical recommendations for data merging: (i) under low Missingness in the covariates, missForest is the optimal choice, delivering the highest performance regardless of the missing data mechanism; (ii) under High Missingness, OTrecod and missMDA provide the most robust and accurate results; (iii) when a method is required that won’t need an imputation step of the covariates before variable recoding, OTrecod is the better choice; (iv) however, if the analytical pipeline strictly requires the imputation of covariates before recoding, missMDA should be the preferred algorithm.

Our empirical validation is based on the National Child Development Study (NCDS). While robust, the specific structural characteristics and variable typologies of this dataset may not generalise to other datasets that feature higher dimensionality or different noise levels. Real-world data often exhibit unpredictable, complex interactions and missing data patterns that may not align perfectly with our controlled experimental conditions.

The scope of this study primarily focused on specific covariate-outcome relationships and the recoding of categorical information. Future research should investigate the generalizability of these findings across a wider variety of continuous distributions and highly heterogeneous real-world datasets to further solidify these FAIR data principles. To achieve this, we will apply this approach to microbiome datasets such as BacDive (Schober *et al.* 2025) and Omnicrobe (Dérozier *et al.* 2023). We also plan to leverage the OpenHSR dataset (N’kam Suguem and Lafargue 2025) to investigate the variable recoding problem in the context of cross-regional nutritional labels, specifically by harmonising this data with the Open Food Facts database.

## Author contributions

Flore N’kam suguem (Conceptualization [Lead], Data curation [Lead], Formal analysis [Lead], Investigation [Lead], Software [Lead], Visualization [Lead], Writing—original draft [Equal], Writing—review & editing [Equal]), Sébastien Déjean (Conceptualization [Equal], Funding acquisition [Equal], Methodology [Equal], Project administration [Equal], Supervision [Equal], Validation [Equal], Writing—original draft [Equal], Writing—review & editing [Equal]), Philippe Saint Pierre (Conceptualization [Equal], Funding acquisition [Equal], Methodology [Equal], Project administration [Equal], Supervision [Equal], Validation [Equal], Writing—original draft [Equal], Writing—review & editing [Equal]), and Nicolas Savy (Conceptualization [Equal], Funding acquisition [Equal], Methodology [Equal], Project administration [Equal], Supervision [Equal], Validation [Equal], Writing—original draft [Equal], Writing—review & editing [Equal])

## Supplementary Material

btag594_Supplementary_Data

## Data Availability

The National Child Development Study dataset is included in the R package OTrecod from the Comprehensive R Archive Network (CRAN): https://cran.r-project.org.

## References

[btag594-B1] Allison PD. Missing Data. Quantitative Applications in the Social Sciences. SAGE Publications, Inc., 2002. 10.4135/9781412985079

[btag594-B2] Alwateer M , Atlam E-S, Abd El-Raouf MM et al Missing data imputation: a comprehensive review. JCC 2024;12:53–75. 10.4236/jcc.2024.1211004

[btag594-B3] Borowicc S , Souza SNA. Heterogeneous data integration: a literature scope review. In: *Proceedings of the 26th International Conference on Enterprise Information Systems (ICEIS 2024) SCITEPRESS Portugal*, p. 189–200, 2024. 10.5220/0012551000003690

[btag594-B4] Castanedo F. A review of data fusion techniques. ScientificWorldJournal 2013;2013:704504. 10.1155/2013/704504 24288502 PMC3826336

[btag594-B5] Courty N , Flamary R, Rakotomamonjy A et al Optimal transport for domain adaptation. In: *Proceedings of the NIPS 2017 Workshop on Optimal Transport and Machine Learning*, Montréal, Canada, 2017. 10.1109/TPAMI.2016.2615921 27723579

[btag594-B6] Cover TM , Hart PE. Nearest neighbor pattern classification. IEEE Trans Inform Theory 1967;13:21–7. 10.1109/TIT.1967.1053964

[btag594-B7] Dempster AP , Laird NM, Rubin DB. Maximum likelihood from incomplete data via the em algorithm. J R Stat Soc Ser B Methodol 1977;39:1–22. http://www.jstor.org/stable/2984875

[btag594-B8] Donders AR , van der Heijden GJ, Stijnen T et al Review: a gentle introduction to imputation of missing values. J Clin Epidemiol 2006;59:1087–91. 10.1016/j.jclinepi.2006.01.014 16980149

[btag594-B9] Dérozier S , Bossy R, Deléger L et al Omnicrobe, an open-access database of microbial habitats and phenotypes using a comprehensive text mining and data fusion approach. PLoS One 2023;18:e0272473. 10.1371/journal.pone.0272473 36662691 PMC9858090

[btag594-B10] Gares V , Dimeglio C, Guernec G et al On the use of optimal transportation theory to recode variables and application to database merging. Int J Biostat 2019;16. 10.1515/ijb-2018-0106 31527293

[btag594-B11] Garès V , Omer J. Regularized optimal transport of covariates and outcomes in data recoding. J Am Stat Assoc 2022;117:320–33. 10.1080/01621459.2020

[btag594-B12] Guernec G , Gares V, Omer J et al OTrecod: an R package for data fusion using optimal transportation theory. R J 2023;14:195–222. 10.32614/RJ-2023-006

[btag594-B13] Kim J, Tae D, Seok J. A Survey of Missing Data Imputation Using Generative Adversarial Networks. In: *2020 International Conference on Artificial Intelligence in Information and Communication (ICAIIC)*, *Fukuoka, Japan*, 2020, pp. 454–6. 10.1109/ICAIIC48513.2020.9065044

[btag594-B14] Josse J , Husson F. missMDA: a package for handling missing values in multivariate data analysis. J Stat Soft 2016;70:1–31.

[btag594-B15] Little RJ. Missing data analysis. Ann Rev Clin Psychol 2024;20:149–73. 10.1146/annurev-clinpsy-080822-051727 38346291

[btag594-B16] Mayer I , Sportisse A, Josse J et al R-miss-tastic: a unified platform for missing values methods and workflows. 2022. 10.32614/RJ-2022-040

[btag594-B17] Mohamed S , Farah K, Lotfy A et al Knowledge graphs: The future of data integration and insightful discovery. In: Advanced Research Trends in Sustainable Solutions, Data Analytics, and Security, 99–146. Hershey, PA, USA: IGI Global Scientific Publishing, 2025. 10.4018/979-8-3693-7117-6.ch005

[btag594-B18] Monge G. Mémoire Sur La Théorie Des Déblais Et Des Remblais. Paris, France: Imprimerie royale, 1781.

[btag594-B19] N’kam Suguem F , Lafargue V. Open health star rating (openhsr) (version v1). Data set. 2025. 10.5281/zenodo.17469191.

[btag594-B20] Peyré G , Cuturi M. Computational optimal transport: with applications to data science. Found Trends Mach Learn 2019;11:355–607. 10.1561/2200000073

[btag594-B21] Putrama IM , Martinek P. Heterogeneous data integration: challenges and opportunities. Data Brief 2024;56:110853. 10.1016/j.dib.2024.110853 39286416 PMC11402636

[btag594-B22] Robin X , Turck N, Hainard A et al pROC: an open-source package for R and S + to analyze and compare roc curves. BMC Bioinformatics 2011;12:77. 21414208 10.1186/1471-2105-12-77 PMC3068975

[btag594-B23] Rubin DB. Inference and missing data. Biometrika 1976;63:581–92. 10.2307/2335739

[btag594-B24] Rubin DB. Multiple Imputation for Nonresponse in Surveys. Wiley Series in Probability and Statistics. USA: John Wiley & Sons, Inc., 1987. 10.1002/9780470316696

[btag594-B25] Schafer JL. Analysis of Incomplete Multivariate Data, 1st edn. New York: Chapman and Hall/CRC, 1997. 10.1201/9780367803025

[btag594-B26] Schober I , Koblitz J, Sardà Carbasse J et al Bacdive in 2025: the core database for prokaryotic strain data. Nucleic Acids Res 2025;53:D748–56. 10.1093/nar/gkae959 39470737 PMC11701647

[btag594-B27] Schouten RM , Lugtig P, Vink G. Generating missing values for simulation purposes: a multivariate amputation procedure. J Stat Comput Simul 2018;88:2909–30. 10.1080/00949655.2018.1491577

[btag594-B28] Stekhoven DJ , Bühlmann P. Missforest—non-parametric missing value imputation for mixed-type data. Bioinformatics 2012;28:112–8. 22039212 10.1093/bioinformatics/btr597

[btag594-B29] Suciu D. Probabilistic databases for all. In: Proceedings of the 2020 ACM SIGMOD International Conference on Management of Data, p. 2493–508. New York, NY: Association for Computing Machinery, 2020. 10.1145/3318464.3389129

[btag594-B30] van Buuren S. Flexible Imputation of Missing Data, 2nd edn. New York, NY: Chapman and Hall/CRC, 2018. 10.1201/9780429492259

[btag594-B31] van Buuren S , Groothuis-Oudshoorn K. mice: Multivariate imputation by chained equations in R. J Stat Soft 2011;45:1–67. 10.18637/jss.v045.i03

