# Supplementary material for: Comparing optimal transport and machine learning approaches for databases merging in scenarios involving missing data in covariates. Application to medical research
Source: Bioinformatics. 2026 Aug 8;42(8):btag594. doi: 10.1093/bioinformatics/btag594 (PMC13481674; doi:10.1093/bioinformatics/btag594)
Supplement: Supplementary file 1 [file btag594_supplementary_data.pdf]

Comparing Optimal Transport and Machine Learning Approaches for Data Merging Under Missing Covariate Scenarios: Application to Medical Research
Supplementary Materials

Flore N'kam Suguem [1,2,4,*], Sébastien Déjean [1,2], Philippe Saint Pierre [1,2], and Nicolas Savy [1,3 *]

[1]Institut de Mathématiques de Toulouse, UMR521, Université de Toulouse, CNRS, France
[2]Université de Toulouse UT, F-31062 Toulouse Cedex 9, France
[3]Université Toulouse Jean-Jaurès UT2J, F-31058 Toulouse, France
[4]Department of Physics and Astronomy, University of Bologna, 40127 Bologna, Italy
[*]Corresponding author: flore.n_kam_suguem@math.univ-toulouse.fr

July 17, 2026

1 Code and Packages

Algorithm 1 OTrecod Pipeline

Require: Two databases $A = \{1, \ldots, n_A\}$ and $B = \{1, \ldots, n_B\}$, sharing covariates X; target Y observed in A only, target Z observed in B only.

Parameters: Distance metric $\textsc{distance} \in \{\text{Euclidean}, \text{Manhattan}, \text{Gower}, \text{Hamming}\}$; algorithm choice $\textsc{algo} \in \{\text{outcome}, \text{r-outcome}, \text{joint}, \text{r-joint}\}$; relaxation $\alpha \geq 0$; regularisation $\lambda \geq 0$.

Ensure: Completed databases A_{OT} (with predicted $\hat{Z}$) and/or B_{OT} (with predicted $\hat{Y}$).

Step 1: Pre-processing

Harmonise A and B using $\textsc{merge\_dbs}$: align variable types and levels; discard incompatible covariates; handle missing values. Outputs merged `DB_READY`

Select matching variables X via $\textsc{select\_pred}$: compute Spearman / Cramér's V associations; detect collinearity (Farrar–Glauber test); optionally run conditional random forest. Retains parsimonious subset

Step 2: Empirical estimators

Compute marginal estimators: $\hat{\mu}^{Y_A}_{n_A,y} = \frac{1}{n_A} \sum_{i \in A} \mathbf{1}_{\{Y_i = y\}}, \quad \hat{\mu}^{Z_B}_{n_B,z} = \frac{1}{n_B} \sum_{j \in B} \mathbf{1}_{\{Z_j = z\}}$.

Estimate the cost matrix for $\kappa_{n,y,z} \neq 0$:

$$\hat{c}_{n,y,z} = \frac{1}{\kappa_{n,y,z}} \sum_{i \in A} \sum_{j \in B} \mathbf{1}_{\{Y_i = y,\, Z_j = z\}}\, d(X_i, X_j).$$

Step 3: Optimal transport problem

if $\textsc{algo} \in \{\text{outcome}, \text{r-outcome}\}$ **then**

  Transport marginals of Y and Z by solving the linear program $\hat{\mathcal{P}}^0_n$:

  $\hat{\gamma} = \arg\min_{\gamma \geq 0} \langle \hat{c}_n, \gamma \rangle \quad \text{s.t.} \quad \gamma\, \mathbf{1}_{|\mathcal{Z}|} = \hat{\mu}^{Y_A}_{n_A} + e^Y, \;\; \gamma^\top \mathbf{1}_{|\mathcal{Y}|} = \hat{\mu}^{Z_B}_{n_B} + e^Z$

  with relaxation slack $\|e^Y\|_1, \|e^Z\|_1 \leq \sqrt{\alpha/n}$. Set $\alpha = 0$ for unrelaxed *outcome*

else if $\textsc{algo} \in \{\text{joint}, \text{r-joint}\}$ **then**

  Compute joint estimators $\hat{\mu}^{(X_A,Y_A)}_{n_A}$ and $\hat{\mu}^{(X_B,Z_B)}_{n_B}$.

  Transport joint distributions by solving the linear program $\hat{\mathcal{P}}_n$:

  $\hat{\gamma}_{x,y,z} = \arg\min_{\gamma \geq 0} \langle \hat{c}_n, \gamma \rangle \quad \text{s.t.} \quad \sum_z \gamma_{x,y,z} = \hat{\mu}^{(X_A,Y_A)}_{n_A,x,y} + e^{XY}_{x,y}, \;\; \sum_y \gamma_{x,y,z} = \hat{\mu}^{(X_B,Z_B)}_{n_B,x,z} + e^{XZ}_{x,z}$

  if $\lambda > 0$ **then** *r-joint* regularisation

    Add L_1 regularisation term $\lambda \sum_{\{x_i,x_j\} \in \mathcal{E}_X} w_{ij} \sum_{y,z} r^+_{ij,y,z}$ to the objective ($\hat{\mathcal{P}}^R_n$).

  end if

end if

Step 4: Individual predictions

Estimate conditional distribution: $\tilde{\mu}^{Z_A|X_A=x,\,Y_A=y}_{n_A,z} = \frac{\hat{\gamma}_{x,y,z}}{\hat{\mu}^{(X_A,Y_A)}_{n_A,x,y}}$.

Predict individual values: $\hat{z}^A_i = \arg\max_{z \in \mathcal{Z}} \tilde{\mu}^{Z_A|X_A=x_i,\,Y_A=y_i}_{n_A,z}$.

Store predictions in `OTpred` column of A_{OT} and/or B_{OT}. Via `sequential` or `optimal`

Step 5: Validation

Assess fusion quality with $\textsc{verif\_OT}$:

- Cramér's V and Spearman ρ between predicted and observed values.
- Hellinger distance to check $Y_A \overset{d}{=} Y_B$: $d_{\text{Hell}}(Y_A, \hat{Y}_B) = \left(\frac{1}{2} \sum_y \left(\sqrt{\hat{\mu}^{Y_A}_{n_A,y}} - \sqrt{\hat{\mu}^{Y_B}_{n_B,y}}\right)^2\right)^{1/2}$.
- Stability: trimmed mean of $P(\hat{Z} = \hat{z}_i \mid Y = y_i, X = x_i)$.

if quality criteria unsatisfactory **then**

  Increase α (relaxation) and/or λ (regularisation) and **return to Step 3**.

end if

return A_{OT}, B_{OT}

The following packages are used to get the results described in this manuscript: **Software:** All analyses were conducted in R (version 4.4.1) using the following packages:

- OTrecod (version 0.1.2)
- mice (version 3.18.0)
- VIM (version 6.2.2)
- missForest(version 1.6.1)
- missMDA(version 1.21)

All packages are available from the Comprehensive R Archive Network (CRAN): `https://cran.r-project.org`.

OTrecod

- **Type** = R-JOINT
- **Distance** = Hamming
- **Percent closest k-NN** = 100%
- **Relaxation parameter** = YES
- **Relaxation value** = 0.1 (set according to the distribution of our dataset)
- **Regularization term** = 0
- **Aggregation tol cov** = 0.3
- **DB imputed** = BOTH

MICE 3.0

In the `mice` function, the following parameters are specified:

- `method = 'rf'`: Specifies that random forest should be used for imputation.
- `m = 5`: Specifies that 5 imputed datasets should be created.
- `maxit = 5`: Specifies that 5 iterations should be performed.

VIM (kNN)

- **k**: set at 5
- **distVar**: The distance metric typically Euclidean.
- **impVar**: The set of variables to use for imputation (FALSE).

The other parameters were left unchanged as originally set in the R-package VIM. A smaller value of k may overfit, while a larger k might smooth out the imputation.

missForest

- **maxiter**: Set at 5 (the maximum number of iterations to be performed given the stopping criterion).
- **ntree**: Set at 50 (the number of trees to grow in each random forest).

missMDA

- **Method**: `imputeFAMD()` was used to appropriately handle the mixed data types (categorical factors and continuous numeric variables).
- **ncp**: Set at 2 (the number of dimensions/components retained for the Factor Analysis of Mixed Data).

2 Hyperparameters tuning

While standard default values were considered, final algorithm parameters were selected by balancing data-driven tuning metrics across varying sample sizes and R^2 conditions, alongside computational efficiency and established empirical heuristics. For the Optimal Transport framework (OTrecod), we set the proximity penalty to `prox.X` $= 0.10$ and the maximum relaxation term to `maxrelax` $= 0.1$ to balance covariate space preservation with transport cost. For MICE, we utilized Random Forest conditional imputation models for categorical variables, generating $m = 5$ multiple imputations over 5 iterations; although $m = 1$ occasionally yielded better point estimates in specific subsets, $m = 5$ demonstrated competitive stability across both $R^2 = 0.5$ and $R^2 = 0.9$ scenarios and was retained to ensure the robust variance estimation fundamentally required for multiple imputation. For k-Nearest Neighbors (kNN), the neighborhood size was fixed at $k = 5$. While larger neighborhoods ($k = 15$) performed well in the noisier $R^2 = 0.5$ condition, $k = 5$ provided a stronger, more consistent baseline across both explanatory contexts without introducing potential oversmoothing risks. For missForest, forests were restricted to 50 trees ($n_{\text{tree}} = 50$) with a maximum of 5 iterations. Our tuning results consistently showed that 50 trees matched or explicitly outperformed 100 trees across multiple sample sizes and R^2 settings, yielding maximal performance without the added computational cost. Finally, for Factor Analysis of Mixed Data (missMDA), dimensionality reduction was fixed at 2 principal components ($n_{\text{cp}} = 2$); this configuration proved highly robust across both noise levels, maintaining highly competitive precision, Cohen's Kappa, and MAE while avoiding the potential under-representation of the covariate space associated with a single component. The supplementary tables below motivate these choices.

Table 1: Hyperparameter tuning results of imputation methods across different sample sizes ($n \in \{100, 300, 1000\}, F = 1$, and $R^2 = 0.5$)

**Method**	**Param**		$n = 100$			$n = 300$			$n = 1000$		
			**Prec.**	**Kappa**	**MAE**	**Prec.**	**Kappa**	**MAE**	**Prec.**	**Kappa**	**MAE**
MICE	m	1	0.474	0.309	0.563	**0.462**	**0.304**	**0.494**	0.439	0.280	0.468
	m	3	**0.496**	**0.336**	**0.551**	0.449	0.286	0.488	0.436	0.276	0.471
	m	5	0.479	0.315	0.517	0.442	0.281	0.476	**0.441**	**0.284**	0.469
	m	10	0.487	0.330	0.524	0.440	0.277	0.476	0.440	0.280	**0.473**
kNN	k	3	0.495	0.330	0.540	0.481	0.320	0.522	0.451	0.291	0.487
	k	5	0.493	0.320	0.551	0.495	0.331	0.543	0.463	0.299	0.507
	k	10	0.529	0.357	0.590	0.502	0.335	0.558	0.473	0.307	0.522
	k	15	**0.543**	**0.375**	**0.601**	**0.509**	**0.341**	**0.563**	**0.481**	**0.315**	**0.534**
missForest	n_{tree}	10	0.515	0.351	0.563	0.487	0.323	0.530	0.475	0.309	0.526
	n_{tree}	50	**0.524**	0.361	**0.573**	0.485	0.321	0.536	**0.477**	**0.313**	**0.526**
	n_{tree}	100	0.523	**0.362**	0.572	**0.489**	**0.325**	**0.540**	0.468	0.305	0.512
missMDA	n_{cp}	1	**0.556**	**0.384**	**0.613**	**0.528**	0.356	**0.587**	**0.518**	**0.350**	**0.577**
	n_{cp}	2	0.547	0.375	0.602	0.527	**0.359**	0.583	0.508	0.338	0.564
	n_{cp}	3	0.542	0.370	0.596	0.518	0.349	0.574	0.507	0.338	0.564
	n_{cp}	4	0.549	0.380	0.602	0.515	0.346	0.573	0.501	0.333	0.557
	n_{cp}	5	0.536	0.366	0.590	0.512	0.343	0.569	0.497	0.329	0.552

Table 2: Hyperparameter tuning results of imputation methods across different sample sizes ($n \in \{100, 300, 1000\}$) for $F = 0.1$ and $R^2 = 0.9$.

Method	Param		$n = 100$			$n = 300$			$n = 1000$		
			**Prec.**	**Kappa**	**MAE Score**	**Prec.**	**Kappa**	**MAE Score**	**Prec.**	**Kappa**	**MAE Score**
MICE	m	1	**0.523**	**0.190**	**0.600**	0.476	0.142	0.545	**0.496**	**0.158**	**0.570**
	m	3	0.476	0.117	0.554	0.481	0.147	0.550	0.493	0.153	0.568
	m	5	0.491	0.135	0.564	**0.499**	**0.178**	**0.564**	0.491	0.152	0.566
	m	10	0.480	0.120	0.563	0.482	0.151	0.549	0.485	0.141	0.560
kNN	k	3	**0.511**	**0.170**	0.586	**0.502**	**0.174**	0.571	0.490	0.144	0.569
	k	5	0.503	0.153	0.583	0.497	0.160	0.571	**0.496**	**0.152**	0.575
	k	10	0.510	0.159	**0.588**	0.497	0.161	0.573	**0.496**	0.151	**0.576**
	k	15	0.510	0.167	0.587	0.499	0.167	**0.575**	0.495	0.150	0.575
missForest	n_{tree}	10	0.513	0.181	0.588	0.502	0.170	0.574	**0.497**	**0.155**	**0.576**
	n_{tree}	50	**0.526**	**0.195**	**0.601**	0.499	0.166	0.575	**0.497**	0.153	**0.576**
	n_{tree}	100	0.519	0.188	0.594	**0.504**	**0.173**	**0.579**	0.493	0.149	0.574
missMDA	n_{cp}	1	**0.505**	**0.157**	**0.583**	0.486	0.143	0.565	0.494	**0.151**	0.573
	n_{cp}	2	0.502	0.153	0.581	0.491	0.152	**0.569**	**0.495**	0.150	**0.574**
	n_{cp}	3	0.500	0.150	0.579	**0.492**	**0.154**	**0.569**	0.492	0.147	0.572
	n_{cp}	4	0.502	0.154	0.580	0.485	0.142	0.563	0.492	0.146	0.572
	n_{cp}	5	0.500	0.149	0.579	0.486	0.145	0.564	0.493	0.149	0.573

3 Tables of Results

Table 3: Scenario 1: Effect of varying feature magnitude $F \in \{0.1, 1, 9\}$, with Z_L, MMD = CC, AonD = S, $n = 500$, and $R^2 = 0.9$. See Figure 2(a) of the article.

**% F**	**Metric**	**OTrecod**	**MICE**	**kNN**	**missForest**	**missMDA**
0.1	Precision	0.6753 ± 0.0253	0.4985 ± 0.0091	0.5073 ± 0.0085	$\mathbf{0.7770 \pm 0.0171}$	0.7129 ± 0.0362
	Kappa	0.4877 ± 0.0312	0.1677 ± 0.0136	0.1738 ± 0.0137	$\mathbf{0.6288 \pm 0.0261}$	0.5166 ± 0.0621
	MAE Score	0.724 ± 0.026	0.569 ± 0.009	0.581 ± 0.008	$\mathbf{0.815 \pm 0.014}$	0.762 ± 0.030
1	Precision	0.7637 ± 0.0075	0.5342 ± 0.0093	0.5397 ± 0.0095	$\mathbf{0.8310 \pm 0.0103}$	0.7883 ± 0.0115
	Kappa	0.6805 ± 0.0096	0.3629 ± 0.0115	0.3685 ± 0.0115	$\mathbf{0.7653 \pm 0.0138}$	0.6964 ± 0.0161
	MAE Score	0.802 ± 0.006	0.599 ± 0.009	0.606 ± 0.009	$\mathbf{0.859 \pm 0.009}$	0.825 ± 0.010
9	Precision	0.6301 ± 0.0259	0.5997 ± 0.0184	0.6026 ± 0.0187	$\mathbf{0.8009 \pm 0.0111}$	0.7395 ± 0.0076
	Kappa	0.4085 ± 0.0292	0.1999 ± 0.0177	0.2038 ± 0.0166	$\mathbf{0.5774 \pm 0.0293}$	0.3498 ± 0.0176
	MAE Score	0.693 ± 0.021	0.656 ± 0.017	0.660 ± 0.017	$\mathbf{0.835 \pm 0.009}$	0.785 ± 0.006

Pictures/before_2.png

(a) Histogram of the values before categorisation

Pictures/after_2.png

Table 4: Scenario 2: Effect of increasing sample size $n \in \{100, \ldots, 1000\}$, with Z_L, MMD = CC, AonD = S, $F = 1$, and $R^2 = 0.9$. See Figure 2(b) of the article.

n	Metric	OTrecod	MICE	kNN	missForest	missMDA
100	Precision	0.6312 ± 0.0282	0.5327 ± 0.0219	0.5308 ± 0.0203	$\mathbf{0.7969 \pm 0.0209}$	0.7638 ± 0.0194
	Kappa	0.5251 ± 0.0307	0.3578 ± 0.0271	0.3544 ± 0.0247	$\mathbf{0.7175 \pm 0.0270}$	0.6626 ± 0.0266
	MAE Score	0.6806 ± 0.0304	0.6021 ± 0.0195	0.6017 ± 0.0178	$\mathbf{0.8321 \pm 0.0173}$	0.8048 ± 0.0160
200	Precision	0.7100 ± 0.0146	0.5424 ± 0.0162	0.5407 ± 0.0197	$\mathbf{0.7898 \pm 0.0175}$	0.7679 ± 0.0243
	Kappa	0.6161 ± 0.0177	0.3718 ± 0.0200	0.3696 ± 0.0237	$\mathbf{0.7100 \pm 0.0227}$	0.6689 ± 0.0338
	MAE Score	0.7548 ± 0.0133	0.6049 ± 0.0143	0.6055 ± 0.0173	$\mathbf{0.8256 \pm 0.0147}$	0.8082 ± 0.0201
300	Precision	0.7356 ± 0.0132	0.5312 ± 0.0124	0.5411 ± 0.0121	$\mathbf{0.8292 \pm 0.0113}$	0.7715 ± 0.0106
	Kappa	0.6460 ± 0.0172	0.3593 ± 0.0149	0.3703 ± 0.0138	$\mathbf{0.7629 \pm 0.0150}$	0.6724 ± 0.0147
	MAE Score	0.7783 ± 0.0112	0.5972 ± 0.0127	0.6073 ± 0.0122	$\mathbf{0.8585 \pm 0.0095}$	0.8112 ± 0.0088
400	Precision	0.7531 ± 0.0111	0.5486 ± 0.0098	0.5446 ± 0.0100	$\mathbf{0.8380 \pm 0.0106}$	0.8041 ± 0.0109
	Kappa	0.6661 ± 0.0143	0.3815 ± 0.0119	0.3754 ± 0.0118	$\mathbf{0.7746 \pm 0.0143}$	0.7180 ± 0.0154
	MAE Score	0.7934 ± 0.0094	0.6122 ± 0.0089	0.6104 ± 0.0091	$\mathbf{0.8655 \pm 0.0090}$	0.8381 ± 0.0090
500	Precision	0.7598 ± 0.0082	0.5407 ± 0.0109	0.5485 ± 0.0103	$\mathbf{0.8361 \pm 0.0104}$	0.7806 ± 0.0142
	Kappa	0.6740 ± 0.0109	0.3710 ± 0.0127	0.3799 ± 0.0123	$\mathbf{0.7719 \pm 0.0141}$	0.6869 ± 0.0192
	MAE Score	0.7987 ± 0.0067	0.6057 ± 0.0102	0.6140 ± 0.0097	$\mathbf{0.8639 \pm 0.0089}$	0.8187 ± 0.0117
1000	Precision	0.7789 ± 0.0060	0.5387 ± 0.0064	0.5455 ± 0.0071	$\mathbf{0.8493 \pm 0.0050}$	0.7856 ± 0.0073
	Kappa	0.6980 ± 0.0078	0.3683 ± 0.0076	0.3760 ± 0.0083	$\mathbf{0.7896 \pm 0.0067}$	0.6927 ± 0.0101
	MAE Score	0.8155 ± 0.0051	0.6031 ± 0.0053	0.6103 ± 0.0060	$\mathbf{0.8750 \pm 0.0042}$	0.8228 ± 0.0060

Table 5: Scenario 3: Effect of varying signal strength $R^2 \in \{0.2, 0.5, 0.9\}$, with Z_L, MMD = CC, AonD = S, $n = 500$, and $F = 1$. See Figure 2(c) of the article.

R^2	Metric	OTrecod	MICE	kNN	missForest	missMDA
0.2	Precision	0.3685 ± 0.0078	0.3203 ± 0.0095	0.3246 ± 0.0073	0.3905 ± 0.0099	$\mathbf{0.4227 \pm 0.0104}$
	Kappa	0.2469 ± 0.0090	0.1893 ± 0.0107	0.1920 ± 0.0080	0.2657 ± 0.0103	$\mathbf{0.2930 \pm 0.0106}$
	MAE Score	0.3334 ± 0.0093	0.2440 ± 0.0143	0.2579 ± 0.0123	0.3812 ± 0.0145	$\mathbf{0.4483 \pm 0.0146}$
0.5	Precision	0.5605 ± 0.0090	0.4423 ± 0.0069	0.4554 ± 0.0086	0.5853 ± 0.0138	$\mathbf{0.6318 \pm 0.0085}$
	Kappa	0.4366 ± 0.0106	0.2837 ± 0.0075	0.2940 ± 0.0097	0.4572 ± 0.0155	$\mathbf{0.5022 \pm 0.0107}$
	MAE Score	0.6171 ± 0.0084	0.4693 ± 0.0093	0.4918 ± 0.0102	0.6450 ± 0.0135	$\mathbf{0.6925 \pm 0.0072}$
0.9	Precision	0.7621 ± 0.0089	0.5441 ± 0.0097	0.5443 ± 0.0092	$\mathbf{0.8378 \pm 0.0082}$	0.7792 ± 0.0113
	Kappa	0.6785 ± 0.0116	0.3761 ± 0.0116	0.3751 ± 0.0113	$\mathbf{0.7741 \pm 0.0115}$	0.6830 ± 0.0160
	MAE Score	0.8012 ± 0.0077	0.6064 ± 0.0096	0.6079 ± 0.0092	$\mathbf{0.8655 \pm 0.0071}$	0.8175 ± 0.0093

Table 6: Scenario 4: Effect of varying correlation $\rho \in \{0.1, 0.5, 0.7\}$, with Z_L, MMD = CC, AonD = S sample size $n = 500$, $R^2 = 0.9$, and $F = 1$ See Figure 2(d) of the article.

ρ	Metric	OTrecod	MICE	kNN	missForest	missMDA
0.1	Precision	0.5439 ± 0.0084	0.2920 ± 0.0079	0.2899 ± 0.0075	0.5547 ± 0.0074	$\mathbf{0.5634 \pm 0.0087}$
	Kappa	0.4664 ± 0.0098	0.1720 ± 0.0092	0.1696 ± 0.0087	0.4792 ± 0.0086	$\mathbf{0.4903 \pm 0.0097}$
	MAE Score	0.5971 ± 0.0086	0.1112 ± 0.0129	0.1007 ± 0.0139	$\mathbf{0.6143 \pm 0.0069}$	0.6108 ± 0.0112
0.5	Precision	0.5492 ± 0.0060	0.2897 ± 0.0053	0.2914 ± 0.0069	0.5495 ± 0.0076	$\mathbf{0.5659 \pm 0.0081}$
	Kappa	0.4723 ± 0.0071	0.1685 ± 0.0063	0.1707 ± 0.0080	0.4730 ± 0.0091	$\mathbf{0.4930 \pm 0.0089}$
	MAE Score	0.6026 ± 0.0059	0.1157 ± 0.0108	0.1102 ± 0.0118	0.6069 ± 0.0083	$\mathbf{0.6090 \pm 0.0090}$
0.7	Precision	0.5491 ± 0.0063	0.2941 ± 0.0069	0.2898 ± 0.0072	0.5510 ± 0.0068	$\mathbf{0.5685 \pm 0.0076}$
	Kappa	0.4722 ± 0.0075	0.1738 ± 0.0078	0.1689 ± 0.0082	0.4747 ± 0.0079	$\mathbf{0.4959 \pm 0.0085}$
	MAE Score	0.6040 ± 0.0060	0.1155 ± 0.0113	0.1048 ± 0.0104	0.6088 ± 0.0069	$\mathbf{0.6174 \pm 0.0083}$

Table 7: Scenario 5: Effect of increasing sample size $n \in \{100, \ldots, 1000\}$, with Z_{sig}, MMD = CC, AonD = S, $F = 1$, and $R^2 = 0.9$. See Figure 2(e) of the article.

n	**Metric**	**OTrecod**	**MICE**	**kNN**	**missForest**	**missMDA**
100	Precision	0.5070 ± 0.0216	0.2890 ± 0.0185	0.2897 ± 0.0159	$\mathbf{0.5263 \pm 0.0158}$	0.5063 ± 0.0181
	Kappa	0.4227 ± 0.0249	0.1690 ± 0.0213	0.1708 ± 0.0184	$\mathbf{0.4474 \pm 0.0180}$	0.4257 ± 0.0206
	MAE Score	0.5298 ± 0.0252	0.1174 ± 0.0289	0.1182 ± 0.0291	$\mathbf{0.5647 \pm 0.0188}$	0.5397 ± 0.0261
200	Precision	0.5162 ± 0.0103	0.2837 ± 0.0106	0.2843 ± 0.0108	0.5383 ± 0.0137	$\mathbf{0.5393 \pm 0.0131}$
	Kappa	0.4347 ± 0.0119	0.1638 ± 0.0121	0.1650 ± 0.0123	0.4613 ± 0.0158	$\mathbf{0.4639 \pm 0.0147}$
	MAE Score	0.5514 ± 0.0131	0.1040 ± 0.0155	0.1066 ± 0.0183	$\mathbf{0.5876 \pm 0.0156}$	0.5817 ± 0.0145
300	Precision	0.5270 ± 0.0127	0.2846 ± 0.0118	0.2804 ± 0.0115	$\mathbf{0.5533 \pm 0.0093}$	0.5518 ± 0.0087
	Kappa	0.4463 ± 0.0148	0.1629 ± 0.0136	0.1583 ± 0.0132	$\mathbf{0.4777 \pm 0.0107}$	0.4763 ± 0.0098
	MAE Score	0.5726 ± 0.0123	0.1007 ± 0.0178	0.0957 ± 0.0161	$\mathbf{0.6100 \pm 0.0085}$	0.5899 ± 0.0137
400	Precision	0.5195 ± 0.0107	0.2877 ± 0.0074	0.2891 ± 0.0073	0.5393 ± 0.0069	$\mathbf{0.5573 \pm 0.0100}$
	Kappa	0.4380 ± 0.0124	0.1669 ± 0.0085	0.1688 ± 0.0084	0.4617 ± 0.0078	$\mathbf{0.4836 \pm 0.0111}$
	MAE Score	0.5669 ± 0.0130	0.1092 ± 0.0130	0.1026 ± 0.0118	0.5981 ± 0.0078	$\mathbf{0.6012 \pm 0.0141}$
500	Precision	0.5367 ± 0.0073	0.2921 ± 0.0065	0.2923 ± 0.0053	0.5507 ± 0.0098	$\mathbf{0.5673 \pm 0.0060}$
	Kappa	0.4575 ± 0.0085	0.1712 ± 0.0075	0.1715 ± 0.0061	0.4741 ± 0.0113	$\mathbf{0.4943 \pm 0.0066}$
	MAE Score	0.5887 ± 0.0084	0.1201 ± 0.0126	0.1160 ± 0.0114	$\mathbf{0.6115 \pm 0.0093}$	0.6087 ± 0.0099
1000	Precision	0.5514 ± 0.0048	0.2945 ± 0.0048	0.2904 ± 0.0048	0.5492 ± 0.0045	$\mathbf{0.5784 \pm 0.0046}$
	Kappa	0.4747 ± 0.0055	0.1739 ± 0.0056	0.1691 ± 0.0056	0.4722 ± 0.0052	$\mathbf{0.5067 \pm 0.0052}$
	MAE Score	0.6107 ± 0.0048	0.1154 ± 0.0080	0.1075 ± 0.0075	0.6087 ± 0.0055	$\mathbf{0.6264 \pm 0.0083}$

Table 8: Effect of increasing sample size $n \in \{100, \ldots, 1000\}$, with Z_{quad}, AonD = S, $F = 1$, and $R^2 = 0.9$.

n	**Metric**	**OTrecod**	**MICE**	**kNN**	**missForest**	**missMDA**
100	Precision	0.5290 ± 0.0196	0.2743 ± 0.0138	0.2847 ± 0.0155	$\mathbf{0.6077 \pm 0.0220}$	0.5417 ± 0.0236
	Kappa	0.4471 ± 0.0231	0.1511 ± 0.0155	0.1639 ± 0.0178	$\mathbf{0.5408 \pm 0.0257}$	0.4660 ± 0.0267
	MAE Score	0.5314 ± 0.0222	0.0874 ± 0.0211	0.1060 ± 0.0247	$\mathbf{0.6469 \pm 0.0217}$	0.5669 ± 0.0263
200	Precision	0.5443 ± 0.0124	0.2825 ± 0.0112	0.2793 ± 0.0121	$\mathbf{0.6498 \pm 0.0119}$	0.5980 ± 0.0203
	Kappa	0.4664 ± 0.0144	0.1615 ± 0.0129	0.1584 ± 0.0138	$\mathbf{0.5907 \pm 0.0137}$	0.5315 ± 0.0227
	MAE Score	0.5583 ± 0.0138	0.1054 ± 0.0193	0.1036 ± 0.0222	$\mathbf{0.6917 \pm 0.0112}$	0.6221 ± 0.0212
300	Precision	0.5738 ± 0.0114	0.2876 ± 0.0074	0.2964 ± 0.0085	$\mathbf{0.6619 \pm 0.0102}$	0.6094 ± 0.0128
	Kappa	0.5013 ± 0.0132	0.1666 ± 0.0087	0.1772 ± 0.0097	$\mathbf{0.6044 \pm 0.0118}$	0.5434 ± 0.0147
	MAE Score	0.5975 ± 0.0134	0.1075 ± 0.0141	0.1130 ± 0.0134	$\mathbf{0.7012 \pm 0.0099}$	0.6403 ± 0.0145
400	Precision	0.5862 ± 0.0128	0.2946 ± 0.0083	0.3046 ± 0.0083	$\mathbf{0.6648 \pm 0.0073}$	0.6088 ± 0.0127
	Kappa	0.5154 ± 0.0149	0.1741 ± 0.0097	0.1860 ± 0.0096	$\mathbf{0.6075 \pm 0.0084}$	0.5421 ± 0.0147
	MAE Score	0.6142 ± 0.0135	0.1118 ± 0.0119	0.1225 ± 0.0117	$\mathbf{0.7039 \pm 0.0064}$	0.6316 ± 0.0176
500	Precision	0.6015 ± 0.0082	0.2880 ± 0.0082	0.2935 ± 0.0073	$\mathbf{0.6606 \pm 0.0073}$	0.6230 ± 0.0103
	Kappa	0.5334 ± 0.0096	0.1667 ± 0.0094	0.1731 ± 0.0083	$\mathbf{0.6027 \pm 0.0084}$	0.5590 ± 0.0119
	MAE Score	0.6350 ± 0.0083	0.1034 ± 0.0124	0.1150 ± 0.0111	$\mathbf{0.7017 \pm 0.0072}$	0.6506 ± 0.0140
1000	Precision	0.6444 ± 0.0069	0.2955 ± 0.0051	0.2929 ± 0.0053	$\mathbf{0.6768 \pm 0.0045}$	0.6290 ± 0.0109
	Kappa	0.5837 ± 0.0080	0.1753 ± 0.0059	0.1723 ± 0.0061	$\mathbf{0.6215 \pm 0.0060}$	0.5659 ± 0.0126
	MAE Score	0.6826 ± 0.0065	0.1172 ± 0.0058	0.1180 ± 0.0065	$\mathbf{0.7174 \pm 0.0050}$	0.6569 ± 0.0141

Table 9: Scenario 6: Effect of increasing sample size $n \in \{100, \ldots, 1000\}$, with Z_{exp}, AonD $= S$, $F = 1$, and $R^2 = 0.9$. See Figure 2(f) of the article.

n	**Metric**	**OTrecod**	**MICE**	**kNN**	**missForest**	**missMDA**
	Precision	$\mathbf{0.3013 \pm 0.0171}$	0.2810 ± 0.0165	0.2780 ± 0.0121	0.2833 ± 0.0162	0.2633 ± 0.0131
100	Kappa	$\mathbf{0.1847 \pm 0.0195}$	0.1604 ± 0.0196	0.1583 ± 0.0143	0.1659 ± 0.0188	0.1512 ± 0.0140
	MAE Score	$\mathbf{0.1308 \pm 0.0323}$	0.1085 ± 0.0242	0.1033 ± 0.0224	0.1088 ± 0.0265	0.0702 ± 0.0398
	Precision	$\mathbf{0.3065 \pm 0.0124}$	0.2822 ± 0.0123	0.2825 ± 0.0141	0.2953 ± 0.0116	0.2747 ± 0.0095
200	Kappa	$\mathbf{0.1884 \pm 0.0146}$	0.1607 ± 0.0141	0.1615 ± 0.0162	0.1770 ± 0.0137	0.1571 ± 0.0110
	MAE Score	0.1209 ± 0.0206	0.1039 ± 0.0188	0.1047 ± 0.0225	$\mathbf{0.1277 \pm 0.0151}$	0.0630 ± 0.0253
	Precision	0.2859 ± 0.0129	0.2881 ± 0.0086	0.2899 ± 0.0086	$\mathbf{0.2909 \pm 0.0101}$	0.2852 ± 0.0075
300	Kappa	0.1650 ± 0.0145	0.1677 ± 0.0101	0.1698 ± 0.0099	$\mathbf{0.1717 \pm 0.0112}$	0.1679 ± 0.0078
	MAE Score	0.1107 ± 0.0173	0.1116 ± 0.0142	0.1107 ± 0.0131	$\mathbf{0.1174 \pm 0.0171}$	0.1002 ± 0.0231
	Precision	0.2926 ± 0.0076	0.2845 ± 0.0060	0.2888 ± 0.0086	$\mathbf{0.2941 \pm 0.0082}$	0.2885 ± 0.0086
400	Kappa	0.1722 ± 0.0086	0.1628 ± 0.0070	0.1679 ± 0.0100	$\mathbf{0.1744 \pm 0.0095}$	0.1717 ± 0.0090
	MAE Score	0.1160 ± 0.0112	0.1129 ± 0.0098	0.1202 ± 0.0134	$\mathbf{0.1306 \pm 0.0139}$	0.1026 ± 0.0302
	Precision	$\mathbf{0.3015 \pm 0.0081}$	0.2899 ± 0.0076	0.2925 ± 0.0063	0.2944 ± 0.0063	0.2869 ± 0.0074
500	Kappa	$\mathbf{0.1822 \pm 0.0094}$	0.1688 ± 0.0091	0.1719 ± 0.0074	0.1744 ± 0.0073	0.1691 ± 0.0084
	MAE Score	$\mathbf{0.1250 \pm 0.0147}$	0.1167 ± 0.0123	0.1187 ± 0.0100	0.1178 ± 0.0124	0.1085 ± 0.0215
	Precision	$\mathbf{0.3010 \pm 0.0057}$	0.2895 ± 0.0050	0.2939 ± 0.0046	0.2964 ± 0.0060	0.2888 ± 0.0065
1000	Kappa	$\mathbf{0.1817 \pm 0.0069}$	0.1682 ± 0.0059	0.1735 ± 0.0053	0.1767 ± 0.0069	0.1702 ± 0.0069
	MAE Score	0.1293 ± 0.0086	0.1143 ± 0.0070	0.1212 ± 0.0057	$\mathbf{0.1298 \pm 0.0095}$	0.1093 ± 0.0217

Table 10: Scenario 7: Effect of increasing sample size $n \in \{100, \ldots, 1000\}$, with Z_L, $R^2 = 0.8$, MMD = CC, AonD $= D$.

n	**Metric**	**OTrecod**	**MICE**	**kNN**	**missForest**	**missMDA**
	Precision	0.5000 ± 0.0274	0.6152 ± 0.0252	0.6272 ± 0.0242	0.6244 ± 0.0243	$\mathbf{0.6560 \pm 0.0223}$
100	Kappa	0.3317 ± 0.0285	0.4455 ± 0.0308	0.4576 ± 0.0273	0.4520 ± 0.0274	$\mathbf{0.4879 \pm 0.0256}$
	MAE Score	0.5385 ± 0.0332	0.6721 ± 0.0209	0.6846 ± 0.0203	0.6823 ± 0.0211	$\mathbf{0.7101 \pm 0.0186}$
	Precision	0.5192 ± 0.0171	0.5910 ± 0.0160	0.6048 ± 0.0154	0.6090 ± 0.0136	$\mathbf{0.6477 \pm 0.0138}$
200	Kappa	0.3489 ± 0.0170	0.4180 ± 0.0189	0.4315 ± 0.0188	0.4353 ± 0.0160	$\mathbf{0.4766 \pm 0.0170}$
	MAE Score	0.5613 ± 0.0194	0.6445 ± 0.0141	0.6636 ± 0.0131	0.6653 ± 0.0133	$\mathbf{0.7009 \pm 0.0124}$
	Precision	0.5477 ± 0.0139	0.5926 ± 0.0135	0.6000 ± 0.0147	0.6065 ± 0.0134	$\mathbf{0.6500 \pm 0.0111}$
300	Kappa	0.3773 ± 0.0163	0.4227 ± 0.0165	0.4283 ± 0.0175	0.4351 ± 0.0165	$\mathbf{0.4822 \pm 0.0146}$
	MAE Score	0.5938 ± 0.0148	0.6476 ± 0.0126	0.6594 ± 0.0135	0.6663 ± 0.0115	$\mathbf{0.7021 \pm 0.0101}$
	Precision	0.5656 ± 0.0151	0.5888 ± 0.0121	0.6062 ± 0.0126	0.6138 ± 0.0126	$\mathbf{0.6485 \pm 0.0099}$
400	Kappa	0.3961 ± 0.0179	0.4174 ± 0.0151	0.4361 ± 0.0152	0.4432 ± 0.0145	$\mathbf{0.4807 \pm 0.0121}$
	MAE Score	0.6152 ± 0.0148	0.6467 ± 0.0111	0.6654 ± 0.0116	0.6731 ± 0.0113	$\mathbf{0.7024 \pm 0.0090}$
	Precision	0.5677 ± 0.0116	0.5915 ± 0.0116	0.6059 ± 0.0100	0.6164 ± 0.0097	$\mathbf{0.6425 \pm 0.0106}$
500	Kappa	0.3963 ± 0.0127	0.4205 ± 0.0138	0.4345 ± 0.0118	0.4456 ± 0.0114	$\mathbf{0.4713 \pm 0.0129}$
	MAE Score	0.6194 ± 0.0102	0.6458 ± 0.0107	0.6635 ± 0.0089	0.6735 ± 0.0084	$\mathbf{0.6955 \pm 0.0095}$
	Precision	0.5785 ± 0.0084	0.5790 ± 0.0077	0.5931 ± 0.0068	0.6086 ± 0.0088	$\mathbf{0.6440 \pm 0.0068}$
1000	Kappa	0.4067 ± 0.0098	0.4057 ± 0.0091	0.4205 ± 0.0081	0.4361 ± 0.0104	$\mathbf{0.4732 \pm 0.0084}$
	MAE Score	0.6327 ± 0.0081	0.6356 ± 0.0075	0.6512 ± 0.0066	0.6672 ± 0.0076	$\mathbf{0.6974 \pm 0.0059}$

Table 11: Effect of varying cluster distribution (modalities) (K_A, K_B) with Z_L, Total $K = 7$, $n = 500$, and $R^2 = 1$.

(K_A, K_B)	**Metric**	**Baseline**	**OTrecod**	**MICE**	**kNN**	**missForest**	**missMDA**
	Precision	0.3313 ± 0.0036	0.1670 ± 0.0651	0.6803 ± 0.0074	0.7373 ± 0.0067	$\mathbf{0.7739 \pm 0.0071}$	0.6818 ± 0.0064
$(2, 5)$	Kappa	0.0607 ± 0.0300	0.0653 ± 0.0269	0.5592 ± 0.0105	0.6357 ± 0.0092	$\mathbf{0.6861 \pm 0.0097}$	0.5535 ± 0.0079
	MAE Score	0.0952 ± 0.0531	0.0592 ± 0.0387	0.6944 ± 0.0084	0.7720 ± 0.0064	$\mathbf{0.8077 \pm 0.0063}$	0.6660 ± 0.0077
	Precision	0.2736 ± 0.0037	0.0985 ± 0.0508	0.6769 ± 0.0103	0.7304 ± 0.0073	$\mathbf{0.7820 \pm 0.0048}$	0.6403 ± 0.0148
$(3, 4)$	Kappa	0.0064 ± 0.0184	0.0469 ± 0.0207	0.5596 ± 0.0142	0.6319 ± 0.0100	$\mathbf{0.7037 \pm 0.0066}$	0.5162 ± 0.0191
	MAE Score	0.0983 ± 0.0423	0.0106 ± 0.0342	0.7083 ± 0.0107	0.7750 ± 0.0061	$\mathbf{0.8198 \pm 0.0040}$	0.6867 ± 0.0152
	Precision	0.2686 ± 0.0035	0.1630 ± 0.0520	0.6617 ± 0.0097	0.7230 ± 0.0103	$\mathbf{0.7781 \pm 0.0049}$	0.6398 ± 0.0140
$(4, 3)$	Kappa	0.0028 ± 0.0178	0.0442 ± 0.0203	0.5394 ± 0.0132	0.6196 ± 0.0140	$\mathbf{0.6987 \pm 0.0067}$	0.5164 ± 0.0179
	MAE Score	0.0627 ± 0.0381	0.0038 ± 0.0271	0.6930 ± 0.0110	0.7631 ± 0.0089	$\mathbf{0.8165 \pm 0.0040}$	0.6862 ± 0.0158
	Precision	0.3275 ± 0.0043	0.1631 ± 0.0636	0.6785 ± 0.0072	0.7347 ± 0.0056	$\mathbf{0.7701 \pm 0.0061}$	0.6775 ± 0.0090
$(5, 2)$	Kappa	0.0781 ± 0.0266	0.0653 ± 0.0290	0.5570 ± 0.0099	0.6351 ± 0.0075	$\mathbf{0.6824 \pm 0.0083}$	0.5480 ± 0.0117
	MAE Score	0.0723 ± 0.0516	0.0007 ± 0.0426	0.6914 ± 0.0091	0.7598 ± 0.0068	$\mathbf{0.8052 \pm 0.0052}$	0.6647 ± 0.0115

3.1 incomplete case

Table 12: Scenario 8: Effect of MDM = MCAR, with Z_L, MMD = IncC, AonD = S, $n = 100$ to 1000, $F = 1$, $R^2 = 1$, and $P \in \{0.1, 0.5, 0.9\}$. See Figure 3(a) of the article.

% Miss	n	Metric	OTrecod	MICE	kNN	missForest	missMDA
10%	100	Precision	0.6466 ± NA	0.7146 ± 0.0188	0.7500 ± 0.0305	**0.8188 ± 0.0387**	0.7586 ± 0.0175
		Kappa	0.3532 ± NA	0.3300 ± 0.0771	0.3625 ± 0.1147	**0.5557 ± 0.1057**	0.3316 ± 0.1331
		MAE Score	0.7742 ± NA	0.7623 ± 0.0189	0.7934 ± 0.0252	**0.8502 ± 0.0320**	0.8005 ± 0.0145
	200	Precision	0.6953 ± 0.0210	0.7830 ± 0.0203	0.8251 ± 0.0161	**0.8531 ± 0.0167**	0.8047 ± 0.0207
		Kappa	0.3586 ± 0.0277	0.4800 ± 0.0599	0.5836 ± 0.0248	**0.6450 ± 0.0356**	0.4582 ± 0.0797
		MAE Score	0.7655 ± 0.0233	0.8198 ± 0.0169	0.8555 ± 0.0133	**0.8786 ± 0.0138**	0.8386 ± 0.0171
	300	Precision	0.7435 ± 0.0184	0.7807 ± 0.0343	0.8228 ± 0.0107	**0.8495 ± 0.0333**	0.8009 ± 0.0367
		Kappa	0.4303 ± 0.0249	0.4732 ± 0.0684	0.5611 ± 0.0397	**0.6231 ± 0.0703**	0.3853 ± 0.1001
		MAE Score	0.7888 ± 0.0196	0.8193 ± 0.0269	0.8536 ± 0.0088	**0.8756 ± 0.0275**	0.8355 ± 0.0303
	400	Precision	0.7470 ± 0.0190	0.8156 ± 0.0163	0.8372 ± 0.0117	**0.8806 ± 0.0134**	0.8376 ± 0.0161
		Kappa	0.4428 ± 0.0529	0.5643 ± 0.0411	0.6048 ± 0.0487	**0.7017 ± 0.0502**	0.5559 ± 0.1140
		MAE Score	0.7950 ± 0.0175	0.8472 ± 0.0140	0.8655 ± 0.0097	**0.9013 ± 0.0111**	0.8658 ± 0.0133
	500	Precision	0.7403 ± 0.0178	0.7585 ± 0.0159	0.8131 ± 0.0266	**0.8580 ± 0.0228**	0.8096 ± 0.0233
		Kappa	0.4214 ± 0.0514	0.4357 ± 0.0309	0.5485 ± 0.0782	**0.6567 ± 0.0627**	0.4403 ± 0.0934
		MAE Score	0.7870 ± 0.0132	0.8007 ± 0.0126	0.8458 ± 0.0221	**0.8829 ± 0.0193**	0.8426 ± 0.0193
	1000	Precision	0.6991 ± 0.1538	0.7819 ± 0.0158	0.8537 ± 0.0102	**0.8839 ± 0.0141**	0.8078 ± 0.0190
		Kappa	0.4204 ± 0.1079	0.4858 ± 0.0129	0.6536 ± 0.0243	**0.7127 ± 0.0289**	0.4145 ± 0.0468
		MAE Score	0.8152 ± 0.0099	0.8202 ± 0.0127	0.8791 ± 0.0084	**0.9060 ± 0.0099**	0.8412 ± 0.0157
50%	100	Precision	0.6473 ± NA	0.6147 ± 0.0639	0.6563 ± 0.0271	0.6944 ± 0.0471	**0.7193 ± 0.0405**
		Kappa	0.1612 ± NA	0.1246 ± 0.1570	0.1421 ± 0.0891	0.2144 ± 0.0983	**0.3477 ± 0.1163**
		MAE Score	0.7129 ± NA	0.6837 ± 0.0512	0.7160 ± 0.0224	0.7474 ± 0.0389	**0.7664 ± 0.0344**
	200	Precision	0.6997 ± 0.0249	0.6738 ± 0.0244	0.6676 ± 0.0183	0.7007 ± 0.0283	**0.7120 ± 0.0250**
		Kappa	0.2569 ± 0.0479	0.2532 ± 0.0727	0.1857 ± 0.0524	0.2812 ± 0.0639	**0.2933 ± 0.0897**
		MAE Score	0.7501 ± 0.0269	0.7276 ± 0.0209	0.7253 ± 0.0151	0.7526 ± 0.0234	**0.7611 ± 0.0222**
	300	Precision	0.6954 ± 0.0303	0.6822 ± 0.0111	0.7052 ± 0.0151	0.7122 ± 0.0165	**0.7486 ± 0.0234**
		Kappa	0.2900 ± 0.0955	0.2395 ± 0.0325	0.2390 ± 0.0482	0.2463 ± 0.0398	**0.3128 ± 0.0401**
		MAE Score	0.7446 ± 0.0235	0.7367 ± 0.0105	0.7564 ± 0.0125	0.7636 ± 0.0136	**0.7922 ± 0.0193**
	400	Precision	0.6945 ± 0.0044	0.7068 ± 0.0094	0.6940 ± 0.0268	0.7473 ± 0.0335	**0.7605 ± 0.0220**
		Kappa	0.2987 ± 0.0175	0.3086 ± 0.0487	0.2371 ± 0.0458	0.3207 ± 0.1292	**0.3767 ± 0.0930**
		MAE Score	0.7462 ± 0.0040	0.7555 ± 0.0086	0.7462 ± 0.0226	0.7896 ± 0.0283	**0.8021 ± 0.0182**
	500	Precision	0.7108 ± 0.0059	0.7031 ± 0.0258	0.7026 ± 0.0278	0.7391 ± 0.0292	**0.7564 ± 0.0203**
		Kappa	0.3570 ± 0.0712	0.3049 ± 0.0477	0.2705 ± 0.0422	0.3127 ± 0.0556	**0.3834 ± 0.0431**
		MAE Score	0.7634 ± 0.0080	0.7528 ± 0.0217	0.7522 ± 0.0241	0.7844 ± 0.0241	**0.7979 ± 0.0164**
	1000	Precision	0.7039 ± 0.0209	0.6927 ± 0.0090	0.7117 ± 0.0145	0.7156 ± 0.0142	**0.7674 ± 0.0089**
		Kappa	0.2867 ± 0.0394	0.2996 ± 0.0321	0.2825 ± 0.0289	0.2677 ± 0.0426	**0.4144 ± 0.0213**
		MAE Score	0.7551 ± 0.0172	0.7448 ± 0.0079	0.7612 ± 0.0119	0.7647 ± 0.0119	**0.8071 ± 0.0079**
90%	100	Precision	**0.6840** ± NA	0.5410 ± 0.0445	0.6301 ± 0.0491	0.6555 ± 0.0851	0.6271 ± 0.0967
		Kappa	**0.2139** ± NA	−0.0264 ± 0.1151	0.0301 ± 0.1143	0.1021 ± 0.1635	0.0464 ± 0.1817
		MAE Score	**0.7388** ± NA	0.6277 ± 0.0389	0.6943 ± 0.0406	0.7114 ± 0.0626	0.6918 ± 0.0799
	200	Precision	0.5519 ± 0.1222	0.5694 ± 0.0078	0.6380 ± 0.0512	**0.6475 ± 0.0885**	0.6292 ± 0.0624
		Kappa	0.0722 ± 0.1906	−0.0036 ± 0.0532	0.0496 ± 0.0554	**0.1290 ± 0.2339**	0.0071 ± 0.1079
		MAE Score	0.6198 ± 0.1071	0.6418 ± 0.0088	0.6993 ± 0.0441	**0.7087 ± 0.0731**	0.6920 ± 0.0542
	300	Precision	0.5690 ± 0.2516	0.5756 ± 0.0367	0.6227 ± 0.0216	0.6321 ± 0.0582	**0.6898 ± 0.0443**
		Kappa	0.1004 ± 0.4604	0.0247 ± 0.0990	0.0329 ± 0.0656	0.0087 ± 0.0976	**0.1888 ± 0.0561**
		MAE Score	0.6350 ± 0.2169	0.6452 ± 0.0334	0.6860 ± 0.0188	0.6927 ± 0.0490	**0.7409 ± 0.0336**
	400	Precision	0.6068 ± 0.0465	0.5643 ± 0.0199	0.6041 ± 0.0183	0.5737 ± 0.0524	**0.6726 ± 0.0140**
		Kappa	**0.1677 ± 0.0934**	0.0250 ± 0.0316	0.0407 ± 0.0260	−0.0315 ± 0.0898	0.1080 ± 0.0317
		MAE Score	0.6690 ± 0.0431	0.6327 ± 0.0179	0.6701 ± 0.0156	0.6437 ± 0.0456	**0.7293 ± 0.0115**
	500	Precision	**0.7207 ± 0.0303**	0.6361 ± 0.0342	0.6592 ± 0.0344	0.6748 ± 0.0281	0.6938 ± 0.0543
		Kappa	**0.3519 ± 0.1042**	0.1637 ± 0.0698	0.1173 ± 0.0614	0.1650 ± 0.0398	0.3279 ± 0.1251
		MAE Score	**0.7688 ± 0.0245**	0.6959 ± 0.0296	0.7167 ± 0.0288	0.7289 ± 0.0255	0.7457 ± 0.0445
	1000	Precision	0.6507 ± 0.0248	0.5869 ± 0.0137	0.6295 ± 0.0127	0.6447 ± 0.0207	**0.6910 ± 0.0136**
		Kappa	**0.1915 ± 0.0551**	0.0631 ± 0.0290	0.0649 ± 0.0313	0.0624 ± 0.0406	0.1288 ± 0.0265
		MAE Score	0.7070 ± 0.0212	0.6512 ± 0.0121	0.6914 ± 0.0108	0.7047 ± 0.0175	**0.7442 ± 0.0113**

Table 13: Scenario 9: Effect of MDM = MAR, with Z_L, MMD = IncC, AonD = S, $n = 100$ to 1000, $F = 1$, $R^2 = 1$, and $P \in \{0.1, 0.5, 0.9\}$. See Figure 3(b) of the article.

% Miss	n	Metric	OTrecod	MICE	kNN	missForest	missMDA
10%	100	Precision	—	0.6613 ± 0.0743	0.7100 ± 0.0513	$\mathbf{0.7432 \pm 0.0516}$	0.7122 ± 0.1425
		Kappa	—	0.2202 ± 0.1117	0.2984 ± 0.1215	$\mathbf{0.3742 \pm 0.1350}$	0.2291 ± 0.2275
		MAE Score	—	0.7201 ± 0.0614	0.7603 ± 0.0424	$\mathbf{0.7878 \pm 0.0426}$	0.7621 ± 0.1178
	200	Precision	0.7167 ± 0.0862	0.7940 ± 0.0231	0.7908 ± 0.0259	$\mathbf{0.8483 \pm 0.0320}$	0.8205 ± 0.0280
		Kappa	0.4239 ± 0.1514	0.5327 ± 0.0547	0.5269 ± 0.0679	$\mathbf{0.6483 \pm 0.0787}$	0.4865 ± 0.1022
		MAE Score	0.7555 ± 0.0866	0.8297 ± 0.0205	0.8271 ± 0.0214	$\mathbf{0.8746 \pm 0.0264}$	0.8517 ± 0.0231
	300	Precision	0.7309 ± 0.0384	0.7685 ± 0.0130	0.8148 ± 0.0192	$\mathbf{0.8567 \pm 0.0188}$	0.8183 ± 0.0160
		Kappa	0.4063 ± 0.0760	0.4554 ± 0.0214	0.5465 ± 0.0404	$\mathbf{0.6340 \pm 0.0633}$	0.4312 ± 0.0750
		MAE Score	0.7838 ± 0.0241	0.8091 ± 0.0102	0.8469 ± 0.0159	$\mathbf{0.8816 \pm 0.0155}$	0.8498 ± 0.0132
	400	Precision	0.7772 ± 0.0167	0.7758 ± 0.0135	0.8127 ± 0.0081	$\mathbf{0.8624 \pm 0.0226}$	0.8154 ± 0.0197
		Kappa	0.4874 ± 0.0448	0.4862 ± 0.0365	0.5597 ± 0.0269	$\mathbf{0.6702 \pm 0.0556}$	0.5029 ± 0.0640
		MAE Score	0.8201 ± 0.0131	0.8149 ± 0.0113	0.8452 ± 0.0067	$\mathbf{0.8863 \pm 0.0187}$	0.8474 ± 0.0163
	500	Precision	0.7626 ± 0.0316	0.7793 ± 0.0082	0.8317 ± 0.0254	$\mathbf{0.8801 \pm 0.0118}$	0.8071 ± 0.0195
		Kappa	0.4741 ± 0.0781	0.4876 ± 0.0324	0.6070 ± 0.0544	$\mathbf{0.7078 \pm 0.0328}$	0.4378 ± 0.1037
		MAE Score	0.8062 ± 0.0293	0.8172 ± 0.0077	0.8606 ± 0.0210	$\mathbf{0.9009 \pm 0.0098}$	0.8406 ± 0.0161
	1000	Precision	0.7607 ± 0.0197	0.7846 ± 0.0126	0.8445 ± 0.0095	$\mathbf{0.8828 \pm 0.0131}$	0.8214 ± 0.0164
		Kappa	0.4506 ± 0.0423	0.4997 ± 0.0176	0.6356 ± 0.0212	$\mathbf{0.7155 \pm 0.0312}$	0.4357 ± 0.0396
		MAE Score	0.8002 ± 0.0155	0.8217 ± 0.0105	0.8712 ± 0.0081	$\mathbf{0.9031 \pm 0.0108}$	0.8524 ± 0.0136
50%	100	Precision	—	0.6848 ± 0.0381	0.7021 ± 0.0169	$\mathbf{0.7536 \pm 0.0535}$	0.7506 ± 0.0292
		Kappa	—	0.2266 ± 0.1138	0.2037 ± 0.1382	$\mathbf{0.3874 \pm 0.1199}$	0.3469 ± 0.1241
		MAE Score	—	0.7390 ± 0.0329	0.7538 ± 0.0140	$\mathbf{0.7964 \pm 0.0442}$	0.7939 ± 0.0241
	200	Precision	0.7062 ± 0.0209	0.6955 ± 0.0384	0.6763 ± 0.0347	0.7450 ± 0.0165	$\mathbf{0.7749 \pm 0.0380}$
		Kappa	0.2947 ± 0.2076	0.2831 ± 0.0824	0.2179 ± 0.1013	0.3973 ± 0.0699	$\mathbf{0.4395 \pm 0.0776}$
		MAE Score	0.7640 ± 0.0091	0.7483 ± 0.0336	0.7307 ± 0.0298	0.7904 ± 0.0126	$\mathbf{0.8132 \pm 0.0316}$
	300	Precision	0.7087 ± 0.0567	0.7164 ± 0.0229	0.7209 ± 0.0252	0.7519 ± 0.0184	$\mathbf{0.7843 \pm 0.0180}$
		Kappa	0.2765 ± 0.1298	0.3581 ± 0.0415	0.3141 ± 0.0503	0.3857 ± 0.0855	$\mathbf{0.4687 \pm 0.0371}$
		MAE Score	0.7561 ± 0.0473	0.7669 ± 0.0174	0.7688 ± 0.0198	0.7950 ± 0.0152	$\mathbf{0.8217 \pm 0.0149}$
	400	Precision	0.6976 ± 0.0344	0.7058 ± 0.0203	0.7165 ± 0.0147	0.7332 ± 0.0307	$\mathbf{0.7546 \pm 0.0274}$
		Kappa	0.3445 ± 0.1407	0.2897 ± 0.0521	0.2634 ± 0.0536	0.3415 ± 0.1084	$\mathbf{0.3542 \pm 0.1090}$
		MAE Score	0.7546 ± 0.0318	0.7548 ± 0.0182	0.7653 ± 0.0124	0.7795 ± 0.0254	$\mathbf{0.7960 \pm 0.0215}$
	500	Precision	0.7222 ± 0.0174	0.6951 ± 0.0178	0.7158 ± 0.0241	0.7175 ± 0.0308	$\mathbf{0.7522 \pm 0.0187}$
		Kappa	$\mathbf{0.3653 \pm 0.0918}$	0.2934 ± 0.0245	0.3029 ± 0.0553	0.3071 ± 0.0570	0.2982 ± 0.0353
		MAE Score	0.7710 ± 0.0152	0.7471 ± 0.0140	0.7648 ± 0.0194	0.7665 ± 0.0255	$\mathbf{0.7952 \pm 0.0155}$
	1000	Precision	0.7035 ± 0.0161	0.7092 ± 0.0086	0.7244 ± 0.0111	0.7521 ± 0.0095	$\mathbf{0.7731 \pm 0.0159}$
		Kappa	0.3310 ± 0.0521	0.3286 ± 0.0247	0.3381 ± 0.0068	0.3382 ± 0.0637	$\mathbf{0.3911 \pm 0.0494}$
		MAE Score	0.7550 ± 0.0133	0.7580 ± 0.0074	0.7707 ± 0.0098	0.7950 ± 0.0078	$\mathbf{0.8124 \pm 0.0131}$
90%	100	Precision	—	0.6274 ± 0.0402	0.6420 ± 0.0517	$\mathbf{0.6452 \pm 0.0525}$	0.6329 ± 0.1004
		Kappa	—	$\mathbf{0.1138 \pm 0.0976}$	0.0518 ± 0.0564	0.0180 ± 0.0757	0.0286 ± 0.0951
		MAE Score	—	0.6931 ± 0.0311	0.7024 ± 0.0458	$\mathbf{0.7068 \pm 0.0434}$	0.6937 ± 0.0886
	200	Precision	0.7168 ± 0.0017	0.6099 ± 0.0230	0.6445 ± 0.0585	0.5994 ± 0.0401	$\mathbf{0.7368 \pm 0.0464}$
		Kappa	$\mathbf{0.3633 \pm 0.1957}$	0.0833 ± 0.0188	0.0876 ± 0.1281	-0.0023 ± 0.0755	0.2268 ± 0.1330
		MAE Score	0.7673 ± 0.0126	0.6739 ± 0.0240	0.6965 ± 0.0554	0.6648 ± 0.0378	$\mathbf{0.7825 \pm 0.0383}$
	300	Precision	0.6480 ± 0.0220	0.5638 ± 0.0159	0.6216 ± 0.0140	0.5968 ± 0.0556	$\mathbf{0.6767 \pm 0.0273}$
		Kappa	$\mathbf{0.1577 \pm 0.0252}$	0.0110 ± 0.0360	0.0459 ± 0.0281	0.0280 ± 0.1108	0.1106 ± 0.0545
		MAE Score	0.7184 ± 0.0178	0.6307 ± 0.0145	0.6842 ± 0.0114	0.6646 ± 0.0469	$\mathbf{0.7312 \pm 0.0231}$
	400	Precision	0.5958 ± 0.0274	0.5740 ± 0.0125	0.6064 ± 0.0111	0.6164 ± 0.0227	$\mathbf{0.6647 \pm 0.0189}$
		Kappa	$\mathbf{0.1131 \pm 0.0591}$	0.0349 ± 0.0249	0.0079 ± 0.0157	0.0302 ± 0.0554	0.0863 ± 0.0335
		MAE Score	0.6633 ± 0.0226	0.6420 ± 0.0109	0.6718 ± 0.0095	0.6802 ± 0.0197	$\mathbf{0.7204 \pm 0.0159}$
	500	Precision	0.6393 ± 0.0216	0.5816 ± 0.0139	0.6204 ± 0.0083	0.6078 ± 0.0312	$\mathbf{0.6718 \pm 0.0142}$
		Kappa	$\mathbf{0.1984 \pm 0.0457}$	0.0553 ± 0.0317	0.0479 ± 0.0165	0.0306 ± 0.0610	0.0963 ± 0.0269
		MAE Score	0.6975 ± 0.0193	0.6452 ± 0.0120	0.6837 ± 0.0069	0.6730 ± 0.0269	$\mathbf{0.7278 \pm 0.0120}$
	1000	Precision	0.7171 ± 0.0317	0.6417 ± 0.0171	0.6708 ± 0.0219	0.6547 ± 0.0490	$\mathbf{0.7185 \pm 0.0155}$
		Kappa	0.2740 ± 0.0741	0.1719 ± 0.0314	0.1233 ± 0.0227	0.0956 ± 0.0991	$\mathbf{0.3215 \pm 0.0821}$
		MAE Score	0.7655 ± 0.0262	0.7015 ± 0.0145	0.7270 ± 0.0189	0.7138 ± 0.0414	$\mathbf{0.7670 \pm 0.0127}$

Table 14: Scenario 10: Effect of MDM = MNAR, with Z_L, MMD = IncC, AonD = S, $n = 100$ to 1000, $F = 1$, $R^2 = 1$, and $P \in \{0.1, 0.5, 0.9\}$. See Figure 3(c) of the article.

**% Miss**	n	**Metric**	**OTrecod**	**MICE**	**kNN**	**missForest**	**missMDA**
10%	100	Precision	$0.6500 \pm$ NA	0.7196 ± 0.0383	0.7759 ± 0.0334	0.7857 ± 0.0419	$\mathbf{0.7937 \pm 0.0255}$
		Kappa	$0.3597 \pm$ NA	0.3643 ± 0.0800	$\mathbf{0.4656 \pm 0.0592}$	0.4497 ± 0.1051	0.4093 ± 0.0582
		MAE Score	$0.7282 \pm$ NA	0.7729 ± 0.0328	0.8148 ± 0.0276	0.8279 ± 0.0279	$\mathbf{0.8295 \pm 0.0211}$
	200	Precision	0.7206 ± 0.0502	0.7338 ± 0.0135	0.8045 ± 0.0137	$\mathbf{0.8292 \pm 0.0149}$	0.7845 ± 0.0182
		Kappa	0.4562 ± 0.1223	0.3852 ± 0.0309	0.5438 ± 0.0384	$\mathbf{0.5968 \pm 0.0372}$	0.3932 ± 0.0469
		MAE Score	0.7897 ± 0.0030	0.7810 ± 0.0112	0.8384 ± 0.0113	$\mathbf{0.8588 \pm 0.0123}$	0.8219 ± 0.0150
	300	Precision	0.7469 ± 0.0156	0.7528 ± 0.0109	0.8056 ± 0.0081	$\mathbf{0.8418 \pm 0.0096}$	0.7941 ± 0.0102
		Kappa	0.4631 ± 0.0240	0.4300 ± 0.0268	0.5348 ± 0.0234	$\mathbf{0.6219 \pm 0.0252}$	0.3908 ± 0.0350
		MAE Score	0.7985 ± 0.0196	0.7959 ± 0.0091	0.8393 ± 0.0067	$\mathbf{0.8695 \pm 0.0081}$	0.8298 ± 0.0084
	400	Precision	0.7236 ± 0.0269	0.7746 ± 0.0129	0.8146 ± 0.0244	$\mathbf{0.8579 \pm 0.0210}$	0.7936 ± 0.0279
		Kappa	0.4037 ± 0.0443	0.4838 ± 0.0380	0.5676 ± 0.0384	$\mathbf{0.6499 \pm 0.0438}$	0.3897 ± 0.0595
		MAE Score	0.7730 ± 0.0245	0.8170 ± 0.0078	0.8468 ± 0.0202	$\mathbf{0.8840 \pm 0.0162}$	0.8294 ± 0.0231
	500	Precision	0.7795 ± 0.0168	0.7980 ± 0.0055	0.8445 ± 0.0124	$\mathbf{0.8709 \pm 0.0152}$	0.8061 ± 0.0289
		Kappa	0.5168 ± 0.0398	0.5380 ± 0.0130	0.6411 ± 0.0187	$\mathbf{0.6990 \pm 0.0276}$	0.4652 ± 0.0924
		MAE Score	0.8179 ± 0.0140	0.8338 ± 0.0051	0.8715 ± 0.0102	$\mathbf{0.8933 \pm 0.0126}$	0.8398 ± 0.0239
	1000	Precision	0.7812 ± 0.0139	0.7925 ± 0.0077	0.8621 ± 0.0056	$\mathbf{0.8946 \pm 0.0083}$	0.8191 ± 0.0207
		Kappa	0.5044 ± 0.0470	0.5177 ± 0.0225	0.6764 ± 0.0130	$\mathbf{0.7475 \pm 0.0218}$	0.4531 ± 0.0730
		MAE Score	0.8173 ± 0.0117	0.8283 ± 0.0063	0.8860 ± 0.0046	$\mathbf{0.9129 \pm 0.0069}$	0.8505 ± 0.0171
50%	100	Precision	0.5069 ± 0.0452	0.5544 ± 0.0408	0.6453 ± 0.0525	0.6880 ± 0.0446	$\mathbf{0.6925 \pm 0.0362}$
		Kappa	0.1225 ± 0.0672	-0.0039 ± 0.0565	0.0870 ± 0.0631	$\mathbf{0.2266 \pm 0.0519}$	0.1755 ± 0.0459
		MAE Score	0.6256 ± 0.0291	0.6289 ± 0.0421	0.7069 ± 0.0434	0.7373 ± 0.0416	$\mathbf{0.7459 \pm 0.0299}$
	200	Precision	$0.7461 \pm$ NA	0.6953 ± 0.0196	0.6787 ± 0.0302	0.7036 ± 0.0179	$\mathbf{0.7293 \pm 0.0242}$
		Kappa	$\mathbf{0.5066} \pm$ NA	0.2958 ± 0.0567	0.2358 ± 0.0543	0.2553 ± 0.0462	0.3286 ± 0.1408
		MAE Score	$\mathbf{0.8001} \pm$ NA	0.7470 ± 0.0147	0.7319 ± 0.0272	0.7550 ± 0.0148	0.7763 ± 0.0200
	300	Precision	0.6906 ± 0.0224	0.7046 ± 0.0093	0.6887 ± 0.0229	0.7354 ± 0.0214	$\mathbf{0.7543 \pm 0.0095}$
		Kappa	0.2885 ± 0.1157	0.2863 ± 0.0458	0.1982 ± 0.0669	0.3101 ± 0.0972	$\mathbf{0.3457 \pm 0.0588}$
		MAE Score	0.7451 ± 0.0225	0.7560 ± 0.0080	0.7427 ± 0.0189	0.7813 ± 0.0177	$\mathbf{0.7969 \pm 0.0079}$
	400	Precision	0.7270 ± 0.0302	0.7018 ± 0.0187	0.7163 ± 0.0239	0.7383 ± 0.0380	$\mathbf{0.7719 \pm 0.0190}$
		Kappa	0.2711 ± 0.0961	0.2930 ± 0.0491	0.2831 ± 0.0787	0.3316 ± 0.1292	$\mathbf{0.4015 \pm 0.0808}$
		MAE Score	0.7760 ± 0.0233	0.7530 ± 0.0177	0.7655 ± 0.0198	0.7837 ± 0.0314	$\mathbf{0.8107 \pm 0.0163}$
	500	Precision	0.7009 ± 0.0195	0.6948 ± 0.0354	0.7017 ± 0.0195	0.7421 ± 0.0364	$\mathbf{0.7539 \pm 0.0222}$
		Kappa	0.2673 ± 0.0761	0.2889 ± 0.0767	0.2517 ± 0.0405	$\mathbf{0.3932 \pm 0.1050}$	0.3688 ± 0.0644
		MAE Score	0.7566 ± 0.0167	0.7465 ± 0.0301	0.7528 ± 0.0160	0.7862 ± 0.0312	$\mathbf{0.7966 \pm 0.0183}$
	1000	Precision	0.7033 ± 0.0093	0.7074 ± 0.0206	0.7103 ± 0.0099	0.7366 ± 0.0357	$\mathbf{0.7659 \pm 0.0043}$
		Kappa	0.2962 ± 0.0531	0.3186 ± 0.0525	0.2716 ± 0.0338	0.3251 ± 0.0798	$\mathbf{0.3650 \pm 0.0544}$
		MAE Score	0.7548 ± 0.0077	0.7577 ± 0.0168	0.7601 ± 0.0078	0.7823 ± 0.0295	$\mathbf{0.8065 \pm 0.0036}$
90%	100	Precision	$0.4988 \pm$ NA	$0.5333 \pm$ NA	$0.6088 \pm$ NA	$\mathbf{0.6326} \pm$ NA	$\mathbf{0.6326} \pm$ NA
		Kappa	$\mathbf{0.0091} \pm$ NA	$-0.1280 \pm$ NA	$-0.0423 \pm$ NA	$-0.0072 \pm$ NA	$-0.0072 \pm$ NA
		MAE Score	$0.6126 \pm$ NA	$0.6071 \pm$ NA	$0.6767 \pm$ NA	$\mathbf{0.6964} \pm$ NA	$\mathbf{0.6964} \pm$ NA
	200	Precision	$\mathbf{0.7071 \pm 0.0395}$	0.6155 ± 0.0382	0.6642 ± 0.0527	0.6864 ± 0.0303	0.6589 ± 0.0630
		Kappa	$\mathbf{0.2264 \pm 0.0491}$	0.0648 ± 0.0791	0.1710 ± 0.0822	0.1431 ± 0.0301	0.2244 ± 0.1770
		MAE Score	$\mathbf{0.7550 \pm 0.0326}$	0.6812 ± 0.0330	0.7217 ± 0.0445	0.7401 ± 0.0256	0.7161 ± 0.0511
	300	Precision	$\mathbf{0.7204 \pm 0.0166}$	0.5812 ± 0.0323	0.6680 ± 0.0262	0.6684 ± 0.0353	0.7009 ± 0.0105
		Kappa	$\mathbf{0.3324 \pm 0.1369}$	0.0690 ± 0.0563	0.1706 ± 0.0594	0.1374 ± 0.0674	0.2755 ± 0.0872
		MAE Score	$\mathbf{0.7696 \pm 0.0196}$	0.6503 ± 0.0218	0.7251 ± 0.0224	0.7248 ± 0.0300	0.7501 ± 0.0079
	400	Precision	$\mathbf{0.7147 \pm 0.0209}$	0.5734 ± 0.0100	0.6054 ± 0.0116	0.6026 ± 0.0299	0.6443 ± 0.0213
		Kappa	$\mathbf{0.2947 \pm 0.0560}$	0.0233 ± 0.0230	0.0147 ± 0.0205	0.0294 ± 0.0590	0.0501 ± 0.0405
		MAE Score	$\mathbf{0.7646 \pm 0.0183}$	0.6403 ± 0.0086	0.6719 ± 0.0101	0.6682 ± 0.0260	0.7043 ± 0.0179
	500	Precision	0.6346 ± 0.0220	0.5778 ± 0.0101	0.6200 ± 0.0093	0.5932 ± 0.0302	$\mathbf{0.6755 \pm 0.0140}$
		Kappa	$\mathbf{0.1718 \pm 0.0491}$	0.0328 ± 0.0248	0.0262 ± 0.0192	-0.0149 ± 0.0557	0.0897 ± 0.0281
		MAE Score	0.6853 ± 0.0183	0.6432 ± 0.0090	0.6831 ± 0.0078	0.6602 ± 0.0258	$\mathbf{0.7302 \pm 0.0118}$
	1000	Precision	0.7155 ± 0.0123	0.6344 ± 0.0233	0.6565 ± 0.0121	0.6532 ± 0.0375	$\mathbf{0.7175 \pm 0.0148}$
		Kappa	$\mathbf{0.2425 \pm 0.0329}$	0.1797 ± 0.0604	0.1492 ± 0.0417	0.1022 ± 0.0876	0.3180 ± 0.1085
		MAE Score	0.7644 ± 0.0105	0.6947 ± 0.0199	0.7151 ± 0.0107	0.7122 ± 0.0313	$\mathbf{0.7657 \pm 0.0118}$

3.2 Imputed case

Table 15: Scenario 11: Effect of MDM = MCAR, with Z_L, MMD = ImpC, AonD = S, $n = 100$ to 1000, $F = 1$, $R^2 = 1$, and $P \in \{0.1, 0.5, 0.9\}$. See Figure 4(a) of the article.

% Miss	n	Metric	OTrecod	MICE	kNN	missForest	missMDA
10%	100	Precision	0.6145 ± 0.1054	0.6817 ± 0.0317	0.7351 ± 0.0485	$\mathbf{0.7766 \pm 0.0455}$	0.7585 ± 0.0849
		Kappa	0.3261 ± 0.1335	0.3061 ± 0.1020	0.4048 ± 0.1165	$\mathbf{0.4938 \pm 0.1448}$	0.4164 ± 0.1729
		MAE Score	0.7320 ± 0.0455	0.7372 ± 0.0322	0.7811 ± 0.0401	$\mathbf{0.8165 \pm 0.0355}$	0.8004 ± 0.0702
	200	Precision	0.5153 ± 0.0601	0.7416 ± 0.0136	0.7971 ± 0.0097	$\mathbf{0.8394 \pm 0.0108}$	0.7999 ± 0.0137
		Kappa	0.2537 ± 0.0708	0.3900 ± 0.0340	0.5206 ± 0.0214	$\mathbf{0.6027 \pm 0.0248}$	0.4152 ± 0.0407
		MAE Score	0.6966 ± 0.0389	0.7871 ± 0.0113	0.8325 ± 0.0081	$\mathbf{0.8674 \pm 0.0089}$	0.8346 ± 0.0113
	300	Precision	0.6675 ± 0.0974	0.7562 ± 0.0107	0.8028 ± 0.0090	$\mathbf{0.8401 \pm 0.0081}$	0.7888 ± 0.0094
		Kappa	0.4219 ± 0.1327	0.4201 ± 0.0269	0.5241 ± 0.0250	$\mathbf{0.6056 \pm 0.0233}$	0.3715 ± 0.0294
		MAE Score	0.7331 ± 0.1638	0.7983 ± 0.0091	0.8369 ± 0.0074	$\mathbf{0.8679 \pm 0.0067}$	0.8255 ± 0.0078
	400	Precision	0.6736 ± 0.0131	0.7554 ± 0.0087	0.8141 ± 0.0079	$\mathbf{0.8533 \pm 0.0064}$	0.7967 ± 0.0081
		Kappa	0.4136 ± 0.0114	0.4264 ± 0.0186	0.5559 ± 0.0191	$\mathbf{0.6402 \pm 0.0170}$	0.3675 ± 0.0289
		MAE Score	0.7371 ± 0.0386	0.7974 ± 0.0072	0.8464 ± 0.0065	$\mathbf{0.8788 \pm 0.0053}$	0.8319 ± 0.0067
	500	Precision	0.7608 ± 0.0091	0.7572 ± 0.0090	0.8214 ± 0.0071	$\mathbf{0.8544 \pm 0.0072}$	0.8030 ± 0.0094
		Kappa	0.4646 ± 0.0204	0.4392 ± 0.0208	0.5747 ± 0.0206	$\mathbf{0.6514 \pm 0.0174}$	0.4068 ± 0.0320
		MAE Score	0.8040 ± 0.0079	0.7995 ± 0.0076	0.8522 ± 0.0059	$\mathbf{0.8800 \pm 0.0060}$	0.8372 ± 0.0078
	1000	Precision	0.7863 ± 0.0045	0.7622 ± 0.0052	0.8615 ± 0.0045	$\mathbf{0.8757 \pm 0.0045}$	0.8066 ± 0.0065
		Kappa	0.5146 ± 0.0119	0.4534 ± 0.0114	0.6752 ± 0.0105	$\mathbf{0.7016 \pm 0.0093}$	0.3976 ± 0.0238
		MAE Score	0.8222 ± 0.0038	0.8026 ± 0.0044	0.8855 ± 0.0037	$\mathbf{0.8973 \pm 0.0037}$	0.8402 ± 0.0054
50%	100	Precision	$0.5073 \pm \text{NA}$	0.6213 ± 0.0178	0.6702 ± 0.0153	0.6837 ± 0.0212	$\mathbf{0.7038 \pm 0.0194}$
		Kappa	$0.1453 \pm \text{NA}$	0.1133 ± 0.0314	0.1567 ± 0.0378	$\mathbf{0.2109 \pm 0.0539}$	0.1948 ± 0.0578
		MAE Score	$0.6953 \pm \text{NA}$	0.6847 ± 0.0151	0.7257 ± 0.0128	0.7371 ± 0.0180	$\mathbf{0.7542 \pm 0.0160}$
	200	Precision	0.6212 ± 0.0146	0.6199 ± 0.0241	0.6653 ± 0.0106	0.6855 ± 0.0145	$\mathbf{0.7150 \pm 0.0168}$
		Kappa	$\mathbf{0.2059 \pm 0.0342}$	0.1209 ± 0.0662	0.1664 ± 0.0335	0.2068 ± 0.0500	0.1945 ± 0.0239
		MAE Score	0.6940 ± 0.0164	0.6845 ± 0.0200	0.7228 ± 0.0088	0.7395 ± 0.0117	$\mathbf{0.7642 \pm 0.0138}$
	300	Precision	0.5276 ± 0.0223	0.6225 ± 0.0142	0.6629 ± 0.0117	0.6834 ± 0.0160	$\mathbf{0.7178 \pm 0.0138}$
		Kappa	0.1343 ± 0.0354	0.1312 ± 0.0306	0.1528 ± 0.0292	$\mathbf{0.2096 \pm 0.0436}$	0.1961 ± 0.0239
		MAE Score	0.6645 ± 0.0195	0.6844 ± 0.0120	0.7202 ± 0.0099	0.7370 ± 0.0136	$\mathbf{0.7663 \pm 0.0116}$
	400	Precision	0.5682 ± 0.0159	0.6324 ± 0.0117	0.6726 ± 0.0077	0.6970 ± 0.0140	$\mathbf{0.7353 \pm 0.0079}$
		Kappa	0.1779 ± 0.0332	0.1557 ± 0.0261	0.1836 ± 0.0223	$\mathbf{0.2312 \pm 0.0373}$	0.2316 ± 0.0155
		MAE Score	0.6587 ± 0.0264	0.6928 ± 0.0101	0.7288 ± 0.0064	0.7492 ± 0.0117	$\mathbf{0.7812 \pm 0.0064}$
	500	Precision	0.6130 ± 0.0323	0.6319 ± 0.0102	0.6697 ± 0.0088	0.7040 ± 0.0109	$\mathbf{0.7302 \pm 0.0078}$
		Kappa	$\mathbf{0.2337 \pm 0.0394}$	0.1528 ± 0.0196	0.1728 ± 0.0190	0.2437 ± 0.0293	0.2154 ± 0.0148
		MAE Score	0.6814 ± 0.0360	0.6916 ± 0.0088	0.7254 ± 0.0072	0.7547 ± 0.0090	$\mathbf{0.7768 \pm 0.0064}$
	1000	Precision	0.6577 ± 0.0072	0.6388 ± 0.0076	0.6799 ± 0.0057	0.7164 ± 0.0074	$\mathbf{0.7374 \pm 0.0043}$
		Kappa	$\mathbf{0.2691 \pm 0.0111}$	0.1802 ± 0.0162	0.2089 ± 0.0152	0.2685 ± 0.0243	0.2305 ± 0.0088
		MAE Score	0.7111 ± 0.0073	0.6965 ± 0.0064	0.7339 ± 0.0048	0.7647 ± 0.0062	$\mathbf{0.7830 \pm 0.0036}$
90%	100	Precision	0.5535 ± 0.0376	0.5410 ± 0.0445	0.6301 ± 0.0491	$\mathbf{0.6555 \pm 0.0851}$	0.6271 ± 0.0967
		Kappa	$\mathbf{0.1418 \pm 0.0053}$	-0.0264 ± 0.1151	0.0301 ± 0.1143	0.1021 ± 0.1635	0.0464 ± 0.1817
		MAE Score	0.6178 ± 0.0653	0.6277 ± 0.0389	0.6943 ± 0.0406	$\mathbf{0.7114 \pm 0.0626}$	0.6918 ± 0.0799
	200	Precision	0.5519 ± 0.1222	0.5694 ± 0.0078	0.6380 ± 0.0512	$\mathbf{0.6475 \pm 0.0885}$	0.6292 ± 0.0624
		Kappa	$\mathbf{0.0722 \pm 0.1906}$	-0.0036 ± 0.0532	0.0496 ± 0.0554	0.1290 ± 0.2339	0.0071 ± 0.1079
		MAE Score	0.6198 ± 0.1071	0.6418 ± 0.0088	0.6993 ± 0.0441	$\mathbf{0.7087 \pm 0.0731}$	0.6920 ± 0.0542
	300	Precision	0.6468 ± 0.0164	0.5774 ± 0.0301	0.6406 ± 0.0270	0.6184 ± 0.0646	$\mathbf{0.6755 \pm 0.0316}$
		Kappa	$\mathbf{0.2382 \pm 0.0454}$	0.0593 ± 0.0591	0.0982 ± 0.0702	0.0866 ± 0.1295	0.1248 ± 0.0682
		MAE Score	0.7032 ± 0.0150	0.6455 ± 0.0256	0.7005 ± 0.0235	0.6817 ± 0.0553	$\mathbf{0.7298 \pm 0.0264}$
	400	Precision	$0.5659 \pm \text{NA}$	0.5990 ± 0.0203	0.6211 ± 0.0167	0.6130 ± 0.0398	$\mathbf{0.6649 \pm 0.0187}$
		Kappa	$0.0898 \pm \text{NA}$	0.0880 ± 0.0469	0.0471 ± 0.0441	0.0197 ± 0.0769	$\mathbf{0.0593 \pm 0.0418}$
		MAE Score	$0.6176 \pm \text{NA}$	0.6635 ± 0.0181	0.6840 ± 0.0143	0.6764 ± 0.0336	$\mathbf{0.7214 \pm 0.0164}$
	500	Precision	0.5799 ± 0.0340	0.5960 ± 0.0175	0.6441 ± 0.0209	0.6249 ± 0.0467	$\mathbf{0.6975 \pm 0.0171}$
		Kappa	$\mathbf{0.1425 \pm 0.0612}$	0.0697 ± 0.0378	0.0813 ± 0.0488	0.0618 ± 0.0968	0.1306 ± 0.0383
		MAE Score	0.6503 ± 0.0272	0.6600 ± 0.0142	0.7035 ± 0.0175	0.6864 ± 0.0398	$\mathbf{0.7486 \pm 0.0142}$
	1000	Precision	0.6343 ± 0.0192	0.5918 ± 0.0167	0.6374 ± 0.0143	0.6453 ± 0.0254	$\mathbf{0.7092 \pm 0.0134}$
		Kappa	$\mathbf{0.2080 \pm 0.0335}$	0.0812 ± 0.0344	0.0857 ± 0.0348	0.0854 ± 0.0509	0.1632 ± 0.0263
		MAE Score	0.6857 ± 0.0198	0.6558 ± 0.0144	0.6981 ± 0.0121	0.7049 ± 0.0215	$\mathbf{0.7593 \pm 0.0112}$

Table 16: Scenario 12: Effect of MDM = MAR, with Z_L, MMD = ImpC, AonD = S, $n = 100$ to 1000, $F = 1$, $R^2 = 1$, and $P \in \{0.1, 0.5, 0.9\}$. See Figure 4(b) of the article.

% Miss	n	Metric	OTrecod	MICE	kNN	missForest	missMDA
10%	100	Precision	0.5970 ± 0.0669	0.6613 ± 0.0743	0.7100 ± 0.0513	**0.7432 ± 0.0516**	0.7122 ± 0.1425
		Kappa	0.3034 ± 0.1173	0.2202 ± 0.1117	0.2984 ± 0.1215	**0.3742 ± 0.1350**	0.2291 ± 0.2275
		MAE Score	0.7201 ± 0.0377	0.7201 ± 0.0614	0.7603 ± 0.0424	**0.7878 ± 0.0426**	0.7621 ± 0.1178
	200	Precision	0.5259 ± 0.0019	0.7509 ± 0.0122	0.8017 ± 0.0118	**0.8458 ± 0.0107**	0.8018 ± 0.0134
		Kappa	0.2940 ± 0.0106	0.4196 ± 0.0306	0.5213 ± 0.0293	**0.6257 ± 0.0252**	0.4169 ± 0.0409
		MAE Score	0.7467 ± 0.0346	0.7952 ± 0.0101	0.8361 ± 0.0098	**0.8726 ± 0.0088**	0.8362 ± 0.0111
	300	Precision	0.5902 ± 0.0558	0.7556 ± 0.0104	0.8027 ± 0.0080	**0.8488 ± 0.0060**	0.7984 ± 0.0101
		Kappa	0.3659 ± 0.0861	0.4135 ± 0.0280	0.5265 ± 0.0196	**0.6234 ± 0.0178**	0.3771 ± 0.0241
		MAE Score	0.8236 ± 0.0069	0.7980 ± 0.0087	0.8369 ± 0.0066	**0.8753 ± 0.0050**	0.8334 ± 0.0083
	400	Precision	0.7018 ± 0.0409	0.7618 ± 0.0076	0.8126 ± 0.0077	**0.8581 ± 0.0077**	0.8039 ± 0.0102
		Kappa	0.4515 ± 0.0530	0.4468 ± 0.0143	0.5522 ± 0.0177	**0.6512 ± 0.0198**	0.4062 ± 0.0378
		MAE Score	0.7480 ± 0.0465	0.8026 ± 0.0063	0.8450 ± 0.0065	**0.8828 ± 0.0064**	0.8379 ± 0.0084
	500	Precision	0.7364 ± 0.0091	0.7592 ± 0.0064	0.8271 ± 0.0056	**0.8641 ± 0.0063**	0.7934 ± 0.0087
		Kappa	0.4977 ± 0.0193	0.4360 ± 0.0127	0.5864 ± 0.0145	**0.6672 ± 0.0153**	0.3686 ± 0.0256
		MAE Score	0.7883 ± 0.0164	0.8007 ± 0.0052	0.8570 ± 0.0046	**0.8878 ± 0.0052**	0.8293 ± 0.0072
	1000	Precision	0.8105 ± 0.0084	0.7631 ± 0.0058	0.8607 ± 0.0041	**0.8755 ± 0.0053**	0.8028 ± 0.0087
		Kappa	0.5983 ± 0.0183	0.4547 ± 0.0132	0.6702 ± 0.0096	**0.6989 ± 0.0127**	0.3824 ± 0.0244
		MAE Score	0.8374 ± 0.0080	0.8033 ± 0.0049	0.8847 ± 0.0035	**0.8971 ± 0.0044**	0.8370 ± 0.0072
50%	100	Precision	0.4837 ± 0.0439	0.6083 ± 0.0193	0.6591 ± 0.0149	0.6593 ± 0.0217	**0.7068 ± 0.0171**
		Kappa	0.0899 ± 0.0846	0.0902 ± 0.0412	0.1361 ± 0.0319	0.1624 ± 0.0574	**0.1850 ± 0.0407**
		MAE Score	0.6033 ± 0.0426	0.6743 ± 0.0174	0.7165 ± 0.0128	0.7169 ± 0.0184	**0.7569 ± 0.0142**
	200	Precision	0.4788 ± 0.0569	0.6193 ± 0.0141	0.6697 ± 0.0135	0.6840 ± 0.0188	**0.7146 ± 0.0195**
		Kappa	0.1440 ± 0.0703	0.1144 ± 0.0285	0.1615 ± 0.0300	**0.1999 ± 0.0464**	0.1878 ± 0.0350
		MAE Score	0.5576 ± 0.0433	0.6829 ± 0.0122	0.7261 ± 0.0111	0.7380 ± 0.0156	**0.7639 ± 0.0160**
	300	Precision	0.4940 ± 0.0547	0.6448 ± 0.0102	0.6771 ± 0.0108	0.7080 ± 0.0112	**0.7280 ± 0.0128**
		Kappa	0.1387 ± 0.0755	0.1843 ± 0.0241	0.1985 ± 0.0256	**0.2758 ± 0.0326**	0.2190 ± 0.0293
		MAE Score	0.6068 ± 0.0444	0.7021 ± 0.0084	0.7307 ± 0.0093	0.7575 ± 0.0096	**0.7742 ± 0.0105**
	400	Precision	0.5652 ± 0.0433	0.6427 ± 0.0098	0.6830 ± 0.0098	0.7099 ± 0.0169	**0.7415 ± 0.0110**
		Kappa	0.1640 ± 0.0407	0.1672 ± 0.0239	0.1971 ± 0.0270	**0.2604 ± 0.0450**	0.2365 ± 0.0239
		MAE Score	0.6521 ± 0.0437	0.7014 ± 0.0083	0.7367 ± 0.0081	0.7589 ± 0.0142	**0.7862 ± 0.0091**
	500	Precision	0.5821 ± 0.0112	0.6364 ± 0.0098	0.6852 ± 0.0094	0.7095 ± 0.0137	**0.7376 ± 0.0074**
		Kappa	0.1955 ± 0.0331	0.1492 ± 0.0233	0.1988 ± 0.0242	**0.2721 ± 0.0432**	0.2262 ± 0.0154
		MAE Score	0.6435 ± 0.0260	0.6950 ± 0.0085	0.7383 ± 0.0078	0.7590 ± 0.0115	**0.7828 ± 0.0060**
	1000	Precision	0.6784 ± 0.0167	0.6391 ± 0.0082	0.6928 ± 0.0049	0.7199 ± 0.0158	**0.7448 ± 0.0057**
		Kappa	**0.2941 ± 0.0293**	0.1785 ± 0.0177	0.2324 ± 0.0148	0.2882 ± 0.0430	0.2424 ± 0.0094
		MAE Score	0.7268 ± 0.0140	0.6971 ± 0.0069	0.7450 ± 0.0041	0.7680 ± 0.0132	**0.7890 ± 0.0047**
90%	100	Precision	—	0.5799 ± 0.0548	0.6057 ± 0.0555	0.5281 ± 0.1179	**0.6311 ± 0.0510**
		Kappa	—	0.0493 ± 0.1259	0.0664 ± 0.0974	−0.0726 ± 0.2769	**0.0874 ± 0.1320**
		MAE Score	0.6126 ± NA	0.6071 ± NA	0.6767 ± NA	0.6964 ± NA	**0.6964** ± NA
	200	Precision	0.5072 ± 0.0622	0.5764 ± 0.0253	0.6069 ± 0.0167	0.6030 ± 0.0520	**0.6628 ± 0.0256**
		Kappa	**0.0741 ± 0.1252**	0.0326 ± 0.0430	0.0078 ± 0.0406	0.0275 ± 0.0949	0.0715 ± 0.0421
		MAE Score	0.5617 ± 0.0909	0.6420 ± 0.0213	0.6721 ± 0.0136	0.6683 ± 0.0445	**0.7194 ± 0.0218**
	300	Precision	0.4365 ± 0.1222	0.5790 ± 0.0132	0.6244 ± 0.0126	0.6168 ± 0.0324	**0.6617 ± 0.0248**
		Kappa	−0.0180 ± 0.1068	0.0293 ± 0.0258	0.0319 ± 0.0297	0.0321 ± 0.0672	**0.0678 ± 0.0476**
		MAE Score	0.5421 ± 0.1178	0.6479 ± 0.0117	0.6873 ± 0.0106	0.6814 ± 0.0283	**0.7193 ± 0.0208**
	400	Precision	0.5324 ± 0.0623	0.5726 ± 0.0106	0.6318 ± 0.0101	0.6145 ± 0.0331	**0.6664 ± 0.0212**
		Kappa	0.0545 ± 0.0881	0.0071 ± 0.0276	0.0402 ± 0.0224	0.0268 ± 0.0705	**0.0715 ± 0.0439**
		MAE Score	0.5865 ± 0.0661	0.6402 ± 0.0089	0.6932 ± 0.0083	0.6770 ± 0.0288	**0.7231 ± 0.0179**
	500	Precision	0.6027 ± 0.0213	0.5850 ± 0.0144	0.6242 ± 0.0087	0.6303 ± 0.0248	**0.6868 ± 0.0101**
		Kappa	**0.1970 ± 0.0239**	0.0544 ± 0.0295	0.0526 ± 0.0223	0.0713 ± 0.0530	0.1177 ± 0.0222
		MAE Score	0.6436 ± 0.0284	0.6505 ± 0.0124	0.6870 ± 0.0076	0.6921 ± 0.0217	**0.7401 ± 0.0084**
	1000	Precision	0.6090 ± 0.0100	0.5854 ± 0.0095	0.6140 ± 0.0068	0.6079 ± 0.0298	**0.6810 ± 0.0113**
		Kappa	**0.1661 ± 0.0249**	0.0603 ± 0.0196	0.0281 ± 0.0127	0.0065 ± 0.0514	0.1142 ± 0.0235
		MAE Score	0.6618 ± 0.0102	0.6508 ± 0.0081	0.6788 ± 0.0057	0.6732 ± 0.0255	**0.7360 ± 0.0095**

Table 17: Scenario 13: Effect of MDM = MNAR, with Z_L, MMD = ImpC, AonD = S, n = 100 to 1000, $F = 1$, $R^2 = 1$, and $P \in \{0.1, 0.5, 0.9\}$. See Figure 4(c) of the article.

% Miss	n	Metric	OTrecod	MICE	kNN	missForest	missMDA
10%	100	Precision	0.5510 ± 0.0557	0.6701 ± 0.0483	0.7818 ± 0.0385	$\mathbf{0.7977 \pm 0.0391}$	0.7323 ± 0.0261
		Kappa	0.2412 ± 0.0732	0.2945 ± 0.0832	0.4735 ± 0.0919	$\mathbf{0.5108 \pm 0.1012}$	0.3269 ± 0.1061
		MAE Score	0.6941 ± 0.0431	0.7377 ± 0.0446	0.8197 ± 0.0318	$\mathbf{0.8340 \pm 0.0338}$	0.7788 ± 0.0216
	200	Precision	0.6861 ± 0.0186	0.7338 ± 0.0135	0.8045 ± 0.0137	$\mathbf{0.8292 \pm 0.0149}$	0.7845 ± 0.0182
		Kappa	0.3853 ± 0.0369	0.3816 ± 0.0309	0.5438 ± 0.0384	$\mathbf{0.5968 \pm 0.0372}$	0.3932 ± 0.0469
		MAE Score	0.7538 ± 0.0197	0.7810 ± 0.0112	0.8384 ± 0.0113	$\mathbf{0.8588 \pm 0.0123}$	0.8219 ± 0.0150
	300	Precision	0.7318 ± 0.0108	0.7528 ± 0.0109	0.8056 ± 0.0081	$\mathbf{0.8418 \pm 0.0096}$	0.7941 ± 0.0102
		Kappa	0.4245 ± 0.0239	0.4300 ± 0.0268	0.5348 ± 0.0234	$\mathbf{0.6219 \pm 0.0252}$	0.3908 ± 0.0350
		MAE Score	0.7854 ± 0.0103	0.7959 ± 0.0091	0.8393 ± 0.0067	$\mathbf{0.8695 \pm 0.0081}$	0.8298 ± 0.0084
	400	Precision	0.7606 ± 0.0066	0.7555 ± 0.0091	0.8156 ± 0.0075	$\mathbf{0.8602 \pm 0.0080}$	0.8006 ± 0.0117
		Kappa	0.4745 ± 0.0161	0.4356 ± 0.0199	0.5597 ± 0.0199	$\mathbf{0.6630 \pm 0.0171}$	0.4175 ± 0.0366
		MAE Score	0.8049 ± 0.0057	0.7983 ± 0.0075	0.8474 ± 0.0063	$\mathbf{0.8846 \pm 0.0067}$	0.8352 ± 0.0097
	500	Precision	0.7670 ± 0.0080	0.7604 ± 0.0070	0.8170 ± 0.0073	$\mathbf{0.8585 \pm 0.0068}$	0.7997 ± 0.0071
		Kappa	0.4800 ± 0.0161	0.4441 ± 0.0143	0.5638 ± 0.0178	$\mathbf{0.6514 \pm 0.0203}$	0.3917 ± 0.0230
		MAE Score	0.8102 ± 0.0066	0.8012 ± 0.0062	0.8487 ± 0.0060	$\mathbf{0.8831 \pm 0.0057}$	0.8345 ± 0.0059
	1000	Precision	0.7890 ± 0.0048	0.7651 ± 0.0068	0.8621 ± 0.0056	$\mathbf{0.8766 \pm 0.0050}$	0.7977 ± 0.0074
		Kappa	0.5219 ± 0.0109	0.4640 ± 0.0143	0.6764 ± 0.0130	$\mathbf{0.7055 \pm 0.0105}$	0.3757 ± 0.0167
		MAE Score	0.8246 ± 0.0038	0.8050 ± 0.0055	0.8860 ± 0.0046	$\mathbf{0.8980 \pm 0.0040}$	0.8328 ± 0.0061
50%	100	Precision	0.5069 ± 0.0452	0.5544 ± 0.0408	0.6453 ± 0.0525	0.6880 ± 0.0446	$\mathbf{0.6925 \pm 0.0362}$
		Kappa	0.1225 ± 0.0672	-0.0039 ± 0.0565	0.0870 ± 0.0631	$\mathbf{0.2266 \pm 0.0519}$	0.1755 ± 0.0459
		MAE Score	0.6256 ± 0.0291	0.6289 ± 0.0421	0.7069 ± 0.0434	0.7373 ± 0.0416	$\mathbf{0.7459 \pm 0.0299}$
	200	Precision	0.6167 ± 0.0161	0.6109 ± 0.0217	0.6582 ± 0.0188	0.6547 ± 0.0338	$\mathbf{0.7059 \pm 0.0296}$
		Kappa	$\mathbf{0.2051 \pm 0.0363}$	0.1204 ± 0.0389	0.1403 ± 0.0614	0.1312 ± 0.0746	0.1867 ± 0.0525
		MAE Score	0.6874 ± 0.0160	0.6778 ± 0.0196	0.7154 ± 0.0155	0.7140 ± 0.0303	$\mathbf{0.7569 \pm 0.0245}$
	300	Precision	0.6552 ± 0.0138	0.6149 ± 0.0129	0.6670 ± 0.0133	0.6759 ± 0.0217	$\mathbf{0.7194 \pm 0.0111}$
		Kappa	$\mathbf{0.2633 \pm 0.0270}$	0.1224 ± 0.0327	0.1736 ± 0.0263	0.1972 ± 0.0522	0.2099 ± 0.0279
		MAE Score	0.7166 ± 0.0142	0.6764 ± 0.0118	0.7231 ± 0.0112	0.7311 ± 0.0188	$\mathbf{0.7678 \pm 0.0093}$
	400	Precision	0.6710 ± 0.0099	0.6313 ± 0.0118	0.6731 ± 0.0123	0.7012 ± 0.0134	$\mathbf{0.7336 \pm 0.0114}$
		Kappa	$\mathbf{0.2715 \pm 0.0207}$	0.1519 ± 0.0226	0.1872 ± 0.0306	0.2536 ± 0.0329	0.2322 ± 0.0236
		MAE Score	0.7283 ± 0.0082	0.6919 ± 0.0102	0.7291 ± 0.0102	0.7526 ± 0.0114	$\mathbf{0.7797 \pm 0.0094}$
	500	Precision	0.6822 ± 0.0089	0.6341 ± 0.0129	0.6816 ± 0.0118	0.7001 ± 0.0152	$\mathbf{0.7396 \pm 0.0125}$
		Kappa	$\mathbf{0.2833 \pm 0.0212}$	0.1605 ± 0.0256	0.1934 ± 0.0256	0.2435 ± 0.0452	0.2384 ± 0.0231
		MAE Score	0.7366 ± 0.0089	0.6938 ± 0.0111	0.7354 ± 0.0101	0.7503 ± 0.0130	$\mathbf{0.7845 \pm 0.0104}$
	1000	Precision	0.7024 ± 0.0059	0.6377 ± 0.0076	0.6878 ± 0.0053	0.7222 ± 0.0081	$\mathbf{0.7414 \pm 0.0053}$
		Kappa	$\mathbf{0.3215 \pm 0.0164}$	0.1839 ± 0.0161	0.2309 ± 0.0127	0.2731 ± 0.0242	0.2409 ± 0.0085
		MAE Score	0.7523 ± 0.0050	0.6955 ± 0.0063	0.7410 ± 0.0045	0.7697 ± 0.0069	$\mathbf{0.7863 \pm 0.0044}$
90%	100	Precision	$0.4988 \pm \text{NA}$	$0.5333 \pm \text{NA}$	$0.6088 \pm \text{NA}$	$\mathbf{0.6326} \pm \text{NA}$	$\mathbf{0.6326} \pm \text{NA}$
		Kappa	$0.0091 \pm \text{NA}$	$-0.1280 \pm \text{NA}$	$-0.0423 \pm \text{NA}$	$-0.0072 \pm \text{NA}$	$\mathbf{-0.0072} \pm \text{NA}$
		MAE Score	$0.6126 \pm \text{NA}$	$0.6071 \pm \text{NA}$	$0.6767 \pm \text{NA}$	$0.6964 \pm \text{NA}$	$\mathbf{0.6964} \pm \text{NA}$
	200	Precision	0.5836 ± 0.0350	0.5957 ± 0.0118	0.6326 ± 0.0121	0.6374 ± 0.0228	$\mathbf{0.6582 \pm 0.0340}$
		Kappa	$\mathbf{0.1584 \pm 0.0611}$	0.0993 ± 0.0364	0.0923 ± 0.0361	0.1152 ± 0.0680	0.0793 ± 0.0476
		MAE Score	0.6517 ± 0.0301	0.6567 ± 0.0108	0.6908 ± 0.0111	0.6950 ± 0.0190	$\mathbf{0.7146 \pm 0.0291}$
	300	Precision	0.6004 ± 0.0246	0.5754 ± 0.0178	0.6214 ± 0.0152	0.6038 ± 0.0361	$\mathbf{0.6635 \pm 0.0109}$
		Kappa	$\mathbf{0.1461 \pm 0.0644}$	0.0351 ± 0.0375	0.0555 ± 0.0220	0.0544 ± 0.0839	0.0875 ± 0.0284
		MAE Score	0.6678 ± 0.0212	0.6402 ± 0.0160	0.6830 ± 0.0131	0.6686 ± 0.0307	$\mathbf{0.7199 \pm 0.0098}$
	400	Precision	0.5905 ± 0.0337	0.5734 ± 0.0100	0.6054 ± 0.0116	0.6026 ± 0.0299	$\mathbf{0.6443 \pm 0.0213}$
		Kappa	$\mathbf{0.0931 \pm 0.0658}$	0.0233 ± 0.0230	0.0147 ± 0.0205	0.0294 ± 0.0590	0.0501 ± 0.0405
		MAE Score	0.6521 ± 0.0283	0.6403 ± 0.0086	0.6719 ± 0.0101	0.6682 ± 0.0260	$\mathbf{0.7043 \pm 0.0179}$
	500	Precision	0.6346 ± 0.0220	0.5778 ± 0.0101	0.6200 ± 0.0093	0.5932 ± 0.0302	$\mathbf{0.6755 \pm 0.0140}$
		Kappa	$\mathbf{0.1718 \pm 0.0491}$	0.0328 ± 0.0248	0.0262 ± 0.0192	-0.0149 ± 0.0557	0.0897 ± 0.0281
		MAE Score	0.6853 ± 0.0183	0.6432 ± 0.0090	0.6831 ± 0.0078	0.6602 ± 0.0258	$\mathbf{0.7302 \pm 0.0118}$
	1000	Precision	0.6696 ± 0.0066	0.5712 ± 0.0084	0.6205 ± 0.0067	0.6085 ± 0.0318	$\mathbf{0.6916 \pm 0.0074}$
		Kappa	$\mathbf{0.2399 \pm 0.0161}$	0.0326 ± 0.0156	0.0324 ± 0.0126	0.0064 ± 0.0485	0.1336 ± 0.0177
		MAE Score	0.7221 ± 0.0067	0.6376 ± 0.0075	0.6837 ± 0.0056	0.6728 ± 0.0275	$\mathbf{0.7444 \pm 0.0063}$
